# Clonal, regionally clustered spread—rather than vertical transmission—explains surges in fluoroquinolone‑resistant *Campylobacter jejuni* in Finnish broilers

**DOI:** 10.1186/s13028-026-00887-0

**Published:** 2026-10-23

**Authors:** Anniina Jaakkonen, Aava Marte, Päivikki Perko-Mäkelä, Hannele Nauholz, Satu Olkkola, Rauni Kivistö

**Affiliations:** 1 Finnish Food Authority, Mustialankatu 3, Helsinki, FI-00790 Finland; 2 Department of Food Hygiene and Environmental Health, Faculty of Veterinary Medicine, University of Helsinki, P.O. Box 66, Helsinki, FI-00014 Finland; 3 Atria Finland Ltd, P.O. Box 900, Atria, Seinäjoki FI-60060 Finland; 4 Animal Health ETT, P.O. Box 221, Seinäjoki, FI-60101 Finland

**Keywords:** Antimicrobial resistance, Campylobacter, Chicken, Ciprofloxacin, Genomic epidemiology, MLST, Surveillance, Tetracycline, Whole-genome sequencing

## Abstract

**Background:**

*Campylobacter jejuni* is a major zoonotic foodborne pathogen, with poultry recognized as an important reservoir for human infection. Although antimicrobial use in Finnish broiler production is minimal, sporadic increases in fluoroquinolone- and tetracycline-resistant *C. jejuni* have been detected. We investigated the population structure, antimicrobial resistance, and transmission pathways of *C. jejuni* in Finnish poultry production.

**Results:**

A total of 333 *C. jejuni* isolates collected from breeder (*n* = 145) and broiler (*n* = 188) flocks during 2014–2019 were characterized using whole-genome sequencing, antimicrobial resistance profiling, multilocus sequence typing (MLST), core-genome MLST (cgMLST), and phylogenomic analysis. Resistance to fluoroquinolones and tetracycline was concentrated in specific years and was strongly associated with particular lineages, especially ST-206 clonal complex (CC), whereas the predominant ST-45 CC was largely susceptible. Genomic clustering identified several resistant clones circulating among geographically proximate broiler farms, indicating regional horizontal dissemination. Resistant isolates from breeders were uncommon, and no genomic evidence supported vertical transmission of resistant strains from breeder to broiler flocks. Instead, resistance peaks were primarily attributable to clonal expansion of successful resistant lineages.

**Conclusions:**

Transient increases in fluoroquinolone- and tetracycline-resistant *C. jejuni* in Finnish broiler production were driven mainly by regional clonal horizontal spread rather than vertical transmission from breeders to broilers. These findings highlight the value of genomic surveillance for detecting and tracking antimicrobial-resistant *Campylobacter* lineages and support the integration of molecular epidemiology into poultry surveillance and control programmes.

**Supplementary Information:**

The online version contains supplementary material available at https://doi.org/10.1186/s13028-026-00887-0.

## Background

*Campylobacter jejuni* is the leading cause of foodborne, zoonotic gastroenteritis in humans globally. The disease is usually self-limiting, but severe cases are treated with antibiotics: macrolides (e.g. azithromycin or erythromycin) and fluoroquinolones (e.g. ciprofloxacin, CIP) are preferred for localized infections in the intestine. Aminoglycosides (e.g. gentamicin) are warranted for systemic infections and bacteraemia, whereas tetracyclines (TET) are not recommended as the first-line treatment due to high resistance rates [1]. Treatment options are limited, however, by increasing antimicrobial resistance of *C. jejuni*, especially against fluoroquinolones [2, 3].

*C. jejuni* is a common commensal in the intestine of many bird and animal species. Various sources of human infection include especially raw or undercooked poultry meat but also animal faeces, contaminated waterbodies, and the environment [1]. *C. jejuni* populations show lineage segregation by host, but also host-generalist genotypes, such as ST-45 (belonging to ST-45 clonal complex, CC), have been recognized by multilocus sequence typing (MLST) [4]. Several studies have associated certain genotypes, such as ST-21 CC and ST-206 CC, with resistance against quinolones in *C. jejuni* isolates from several geographical locations and host species [5–10]. Poultry is generally considered to acquire *C. jejuni* infection horizontally from the environment. A few studies, however, describe also a vertical transmission route from parent to descendant generation via a fertile egg [11–14].

Domestic poultry has been recognized as a major source of infection also in Finland, with a seasonal peak in both human infections and slaughtered broilers during summer months [15]. In Finland, the prevalence of *C. jejuni* and the proportion of antimicrobial-resistant *C. jejuni* isolates in slaughtered broiler flocks have long been below the EU average [2, 16]. Annual resistance rates of 0–2.5% against quinolones (CIP and nalidixic acid, NAL) or TET and 9.2% against only NAL were reported in *C. jejuni* broiler isolates, respectively, before 2013 and in 2013 [17, 18]. In 2014–2019, however, annual resistance rates of *C. jejuni* broiler isolates peaked in certain years with the highest rates reported in 2014: 25.0% against CIP and 17.0% against TET [19]. Finnish quinolone-resistant *C. jejuni* isolates from broilers in 2014 represented distinct genotypes, predominantly ST-122 (ST-206 CC), among fluoroquinolone-resistant *C. jejuni* strains collected from 12 European countries [20]. In the preceding years, the majority of Finnish *C. jejuni* isolates from both broilers and human infections were assigned to ST-45 CC, ST-21 CC, and ST-677 CC [21, 22].

Interestingly, quinolone and TET resistance was detected in *C. jejuni* broiler isolates in certain years in 2014–2019, although Finnish broiler flocks had not been medicated with antibiotics since 2010. Antibiotics had been used, however, to treat annually 2.7–20.2% of breeder flocks during rearing or laying, with the highest proportion of treated flocks in 2014. Penicillin mostly but also amoxicillin, oxytetracycline, and sulfamethoxazole–trimethoprim, were used during this period. In the broiler production chain, quinolones have not been used since 2007 and likely also had not been used before 2007 [23] (Hannele Nauholz, personal communication, 3 July 2024). In 2013–2014, the import of grandparent flocks from Scotland and France was replaced by the import of parent flocks mainly as day-old chicks from Sweden, UK, and the Netherlands (Hannele Nauholz, personal communication, 3 July 2024).

In Finland, broilers are produced on separate farms from breeders according to the all in – all out principle: from eggs to chicks (21 days) in a hatchery and in a broiler house till slaughter age of 35–38 days, followed by cleaning and disinfection of broiler houses during the 14 days between consecutive flocks. Different broiler flocks may be housed simultaneously in different broiler houses on the same farm, and a flock comprises birds that share the same air space. Breeders are slaughtered at the age of 58–65 weeks, after laying for 40 weeks, with a maintenance break of 4–6 weeks between consecutive flocks. Before laying, breeders are typically reared on separate farms from broiler flocks and laying flocks for 18 weeks. Whereas a broiler flock is typically slaughtered in one batch, a breeder flock is slaughtered in more than one batch, divided by compartments, with days or weeks between slaughter batches. Broiler production is based on permanent contracts between meat companies and farmers, which enables traceability in the production chain. There have constantly been three large meat companies, 27 breeder farms (mean size 30,000 laying hens), and 170–180 broiler farms (mean size 80,000 birds) in Finland (Hannele Nauholz, personal communication, 6 May 2026). On average, 6.5% of Finnish broiler batches have been positive for thermotolerant *Campylobacter* spp. (92.1% *C. jejuni*) from June to October, when all slaughter batches have been tested since 2004. In 2016–2019, 26.7% (7.4–49.5% annually) of breeder batches at slaughter were positive for *C. jejuni* (Päivikki Perko-Mäkelä, personal communication, 7 May 2026).

The low prevalence of *C. jejuni*, modest resistance rates, and antibiotics consumption, as well as systematic data collection from both breeder and broiler flocks in 2014–2019, make Finland a valuable model for a genomic epidemiological study. Herein, we analyse whole-genome sequences of 333 *C. jejuni* isolates from Finnish breeder and broiler flocks at slaughter with the aim of assessing associations between genotype and antimicrobial resistance and evaluating the contribution of clonal dissemination, including potential vertical transmission, to episodic increases in resistance.

## Methods

### Bacterial isolates from breeder flocks

Combined caecal samples of 10 birds were taken from breeder flocks at slaughter (age 58–65 weeks) as part of meat companies’ voluntary own-check sampling. All slaughter batches (70–80 annually) were tested in 2014–2019. *Campylobacter* spp. were detected from the samples using direct cultivation onto mCCDA or campyFood agar and microaerobic incubation at 41.5 ± 1 °C for 24–48 h according to ISO 10272-1:2017 by the slaughterhouse or local laboratories. One isolate per flock was sent to the Finnish Food Authority (Helsinki, Finland) for species confirmation by MALDI-TOF (MALDI Biotyper, Bruker Daltonik, Bremen, Germany) and storage at − 70 °C. All *C. jejuni* isolates (*n* = 145, originating from 37 farms) from 2014 to 2019 were subjected to whole-genome sequencing (WGS), which covered all positive breeder flocks over the period.

### Bacterial isolates from broiler flocks

Broiler flocks were sampled in the national control programme and resistance monitoring programme (regulation no. 10/EEO/2007). Combined caecal samples of 10 birds were taken at slaughter (age 35–38 weeks), *Campylobacter* spp. were detected from the samples using mCCDA agar, and species were confirmed as described for breeder flocks. From the year 2014, 64 *C. jejuni* isolates were chosen for WGS to evenly represent the year and different farms. From the years 2015–2019, 125 broiler *C. jejuni* isolates were chosen for WGS with the following criteria: (i) all resistant isolates were included and at least one-third of susceptible isolates, (ii) temporally representative (at least two isolates included from each month when possible), and (iii) more susceptible isolates were chosen for WGS from the periods and farms where resistant isolates were found to maximize the likelihood of detecting resistant and susceptible isolates of the same genotype. The sequenced isolates originated from 89 farms and covered 34–55% of the detected campylobacter-positive flocks each year from 2015 to 2019 and 74% in 2014 (Supplementary Table S1).

### Antimicrobial susceptibility testing of broiler isolates

The antimicrobial susceptibility of broiler *C. jejuni* isolates was studied for ciprofloxacin (CIP), nalidixic acid (NAL), tetracycline (TET), gentamicin (GEN), streptomycin (STR), and erythromycin (ERY) with the broth microdilution method (VetMIC Camp EU, National Veterinary Institute, Uppsala, Sweden), according to the CLSI Vet 01 and manufacturer’s instructions as part of the national campylobacter resistance surveillance included in the FINRES-Vet programme. Details of the annual resistance determination methods and results can be found in the corresponding FINRES-Vet reports [19]. The isolates were classified as wild type (also called susceptible, S, below) or non-wild type (also called resistant, R, below) according to the epidemiological cut-off values by the European Committee on Antimicrobial Susceptibility Testing (https://mic.eucast.org/, accessed 18 March 2026). The minimum inhibitory concentration (MIC) values [mg/L] of R isolates were above 0.5 for CIP, 16 for NAL, 1 for TET, 2 for GEN, 4 for STR, and 4 for ERY.

### Whole-genome sequencing and sequence assembly

*C. jejuni* isolates from both breeders and broilers (*n* = 333) were subjected to WGS using Illumina technology with paired-end reads of 100–250 bp. Breeder flock isolates (*n* = 145) were sequenced using Illumina DNA Prep library preparation kit by Novogene Ltd. (Cambridge, UK) on a NovaSeq instrument (Illumina, USA) resulting in 150 bp paired-end reads. Broiler flock isolates were sequenced using the Nextera XT library preparation kit and HiSeq instrument (Illumina, USA) at the FIMM – Institute for Molecular Medicine (Helsinki, Finland) (59 isolates from the year 2014, 100–150 bp paired-end reads) or Illumina DNA Prep library preparation kit and MiSeq instrument (Illumina, USA) at the Finnish Food Authority (Helsinki, Finland) (129 isolates, 250 bp paired-end reads). All raw reads were randomly subsampled to average coverage of 150-fold before assembly [24].

Raw sequence data were quality-controlled and assembled into contigs using a Docker image of the INNUca pipeline v4.2.2 [25]. Quality control included FastQC and Trimmomatic, retaining reads ≥ 55 bp. INNUca required an estimated coverage of ≥ 15×. TrueCoverage (ReMatCh) used Bowtie2 “--very-sensitive-local” to screen for coverage anomalies. Assemblies were generated with SPAdes using INNUca default settings, including automatic activation of the --isolate option for samples with coverage > 100×. Contigs were filtered using INNUca thresholds (≥ 200 bp, k‑mer coverage ≥ 2, assembly‑mapping coverage ≥ 10×), and assemblies were polished once with Pilon before MLST typing.

### Prediction of antimicrobial resistance

Staramr v0.10.0 [26] implemented in Galaxy Europe [27] was used for in silico prediction of antimicrobial resistance. Draft genome assemblies were screened against the ResFinder [28] (dated 24 May 2022) and PointFinder *Campylobacter* [29] (dated 1 February 2021) databases. For resistance-conferring mutation detection with PointFinder, a minimum sequence identity threshold of 98% and a minimum length overlap threshold of 95% were applied. For acquired antimicrobial resistance gene detection with ResFinder, a minimum sequence identity threshold of 98% and a minimum length overlap threshold of 60% were applied. The lower and upper genome size thresholds for quality metrics were adjusted to 1.5 Mb and 1.9 Mb, respectively.

### Multilocus sequence typing (MLST)

MLST was performed using the Campylobacter MLST database [30] and new allele sequences were submitted to the *Campylobacter jejuni/coli* MLST database (http://pubmlst.org/campylobacter/). Core-genome MLST (cgMLST) analysis was performed using a Docker image of chewBBACA v2.8.5 [31]. To maximize the discriminatory power within our national collection of breeder and broiler isolates, an ad *hoc* cgMLST schema was generated from the assembled genomes using the chewBBACA CreateSchema workflow. The resulting schema comprised 3,728 loci. Paralogous loci were assessed during schema creation, and loci identified as paralogs were excluded from the final schema. Allele calling was subsequently performed against the resulting schema. Core loci were defined as loci detected in at least 95% of the isolates, yielding 1,292 loci. These 1,292 loci constituted the final cgMLST schema used for pairwise allelic distance calculations, minimum spanning tree construction (MSTreeV2), and cluster identification. Minimum spanning trees (MSTreeV2) were visualized using GrapeTree v0.1.8 [32].

### Phylogenomic analysis

Prokka v1.14.6 [33] was used to annotate the draft genomes. Pan-genome analysis was then performed using Roary v3.13.0 [34]. IQ-TREE v2.1.4 [35] model GTR + I+G was used to build a phylogenetic maximum likelihood (ML) tree based on the core genome alignments consisting of genes shared by 99–100% of the strains. The ML tree was rooted at midpoint and annotated using iTOL v6 [36].

### Analysis of production chain information and spatial data

Meat companies provided metadata on *C. jejuni* isolates, including sampling date, identifier of the sampled broiler house, and postal address for the farm of origin. Furthermore, tracking information on the breeder batch associated with each broiler slaughter batch was provided, containing information on the country of origin. The provided data lacked, however, information on the broiler flocks that were imported as hatching eggs from Sweden and Denmark. To calculate straight-line distances in kilometres between farms in a cgMLST cluster, postal addresses of the farms were first geocoded into latitude and longitude points according to the WGS84 coordinate system using the open interface of the National Land Survey, Finland (accessed 18 Apr 2026) and then distances were calculated using the Haversine formula [37].

### Data deposition

The raw sequence data produced in this study (274 isolates) were deposited into the European Nucleotide Archive under project accession number PRJEB113403. Data from the previous study (59 isolates from 2014) are available under project accession number PRJEB23492 [20].

## Results

### Multilocus sequence typing (MLST) of breeder and broiler isolates

**Fig. 1:**
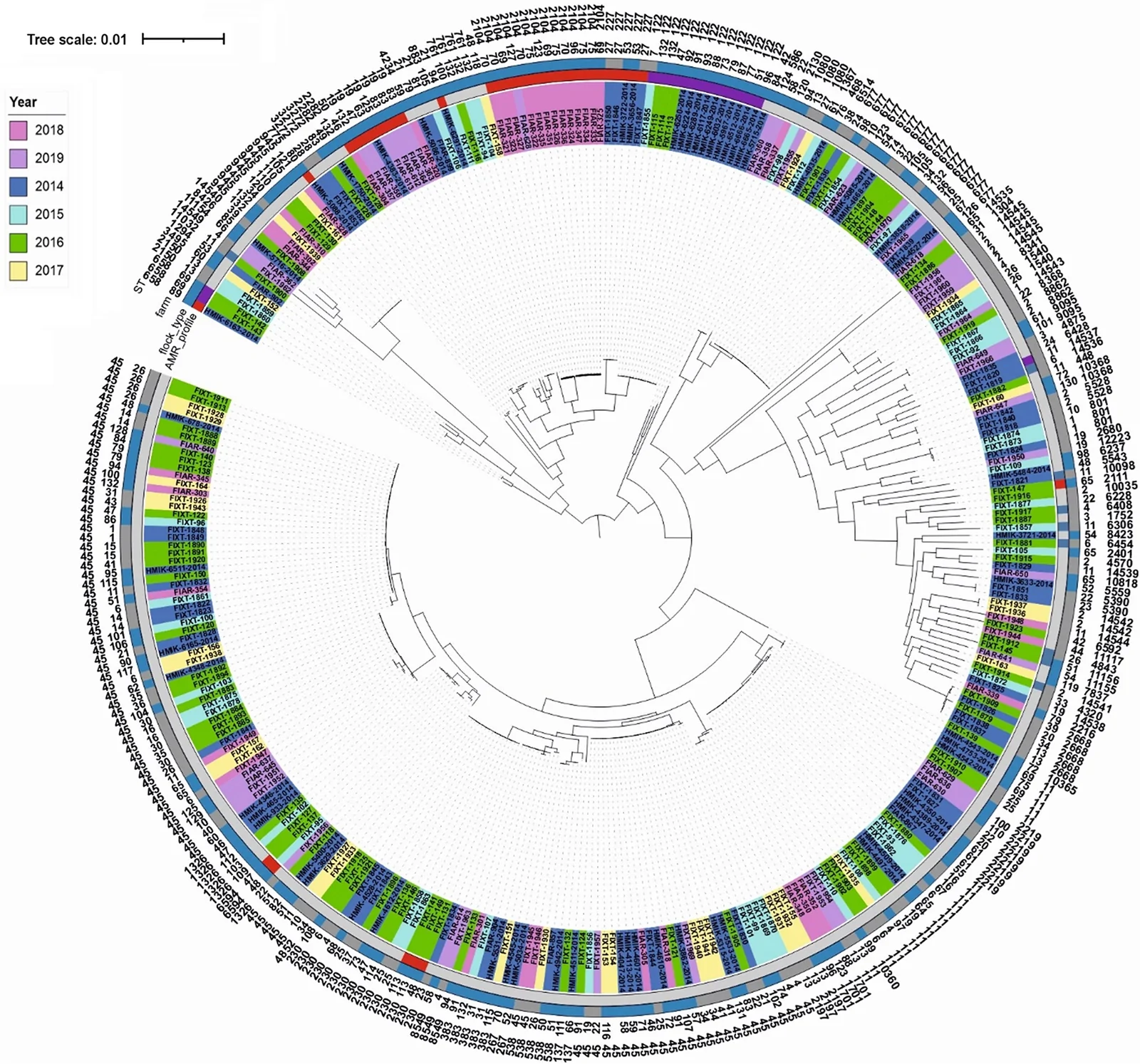
Maximum likelihood phylogeny of 333 *Campylobacter jejuni* isolates. Maximum likelihood tree based on 1,208 core genes and annotated using iTOL. The inner ring consists of the isolate ID coloured by year of isolation. The middle ring shows the genotypic antimicrobial resistance patterns: red denoting CIP-NAL, purple CIP-NAL-TET, blue TET and light grey susceptible isolates. Flock type is denoted by blue and grey for broiler and breeder C. jejuni isolates, respectively. The outermost rings denote anonymized farm ID and ST of C. jejuni isolate, respectively

Altogether, 145 breeder and 188 broiler *C. jejuni* isolates from the years 2014–2019 were sequenced, comprising annually 100% of breeder and 34–74% of broiler isolates (Additional files 1 and 2). The sequenced *C. jejuni* isolates, their origins, and resistance profiles are presented in Additional file 3. MLST typing revealed 15 clonal complexes (CCs) and 95 sequence types (STs), of which 61 STs (64.2%) were represented by single isolates (Fig. 1; Table 1 and Additional file 4). The majority of both breeder (74/145, 51.0%) and broiler (82/188, 43.6%) isolates represented ST-45 CC. ST-45 (ST-45 CC) was the most predominant ST among both breeder (45/145, 31.0%) and broiler (42/188, 22.3%) isolates throughout the monitoring period. Additionally, among breeder isolates, other major STs were ST-11 (ST-45 CC) (12/145, 8.3%), ST-230 (ST-45 CC) (7/145, 4.8%), and ST-677 (ST-677 CC) (7/145, 4.8%), which occurred over several (i.e. more than two) years (Additional file 3). Among broiler isolates, the following STs occurred over several years: ST-677 (ST-677 CC) (10/188, 5.3%), ST-945 (ST-1287 CC) (7/188, 3.7%), ST-1326 (ST-45 CC) (5/188, 2.7%), and ST-383 (ST-283 CC) (5/188, 2.7%), in addition to ST-45. Furthermore, several broiler isolates represented STs belonging to ST-21 CC (14/188, 7.4%) and ST-206 CC (31/188, 16.5%), which occurred over one or two years during the monitoring period. Interestingly, no ST-21 CC was observed among the breeder isolates.

Altogether, 14 STs were represented by both breeder and broiler isolates and belonged to ST-45 CC, ST-206 CC, ST-677 CC, ST-1287 CC, and unassigned CCs (ST-1080 and ST-3272). Breeder and broiler isolates of the same ST mainly originated from different farms, reflecting the Finnish practice of producing breeders and broilers on separate farms (Additional file 3). As an exception in 2017, ST-2197 was observed in *C. jejuni* isolates from breeders during rearing and in broiler isolates from the same farmer.

**Table 1:** Major MLST clonal complexes among breeder and broiler *C. jejuni* isolates

Clonal complex (CC)	Breeder isolates, *n* (%)	Broiler isolates, *n* (%)	Total isolates, *n* (%)
ST-45 CC	74 (51.0)	82 (43.6)	156 (46.8)
ST-206 CC	4 (2.8)	31 (16.5)	35 (10.5)
ST-677 CC	8 (5.5)	10 (5.3)	18 (5.4)
ST-21 CC	0 (0.0)	17 (9.0)	17 (5.1)
ST-1287 CC	2 (1.4)	9 (4.8)	11 (3.3)
Other and unassigned CCs	57 (39.3)	39 (20.7)	96 (28.8)
Total	145 (100)	188 (100)	333 (100)

### Antimicrobial resistance among breeder and broiler isolates

Antimicrobial resistance of breeder isolates was not tested phenotypically. Among the 188 *C. jejuni* broiler isolates, 44 (23.4%) showed phenotypic resistance to CIP, 50 (26.6%) to NAL, and 15 (8.0%) to TET (Table 2). Overall, among the 50 phenotypically resistant broiler isolates, 15 (30.0%) showed combined resistance to CIP-NAL-TET, 29 (58.0%) to CIP-NAL, and 6 (12.0%) to NAL alone. Resistance to CIP and NAL was observed in 2014, 2016, 2018, and 2019, while resistance to TET was observed in only 2014 and 2016 (Additional file 1). No or little resistance was observed in 2015 and 2017.

**Table 2:** Sensitivity and specificity of genetic resistance determinants for predicting phenotypic antimicrobial resistance

Drug(s) tested by MIC determination	Resistance determinant(s)	No. of genotypically resistant isolates^a^	No. of phenotypically resistant isolates	Sensitivity (%)^b^	Specificity (%)^c^
Ciprofloxacin	*gyrA* T86I	43	44	97.7	100.0
Nalidixic acid	*gyrA* T86I/T86A	48	50	96.0	100.0
Tetracycline	*tet(O)* and *tet(O/32/O)*	16	15	100.0	99.4

^a^ For ciprofloxacin, one phenotypically resistant isolate lacked a known resistance-conferring gyrA mutation (T86I or T86A). For nalidixic acid, two phenotypically resistant isolates lacked a known resistance-conferring gyrA mutation. For tetracycline, one isolate (FIXT-145) carried tet(O) but was phenotypically susceptible

^b^ Sensitivity was calculated as the proportion of phenotypically resistant isolates carrying the corresponding resistance determinant

^c^ Specificity was calculated as the proportion of phenotypically susceptible isolates lacking the corresponding resistance determinant

For *C. jejuni* breeder isolates, only genotypic susceptibility data were available, predicting resistance to quinolones in 5/145 isolates (3.4%) and to TET in 10/145 isolates (6.9%). In 2014, 2015, and 2016, respectively, annual genotypic resistance rates of 4/36 (11.1%), 1/23 (4.3%), and 0/42 (0.0%) against quinolones, and 3/36 (8.3%), 2/23 (8.7%), and 5/42 (11.9%) against TET, were predicted among breeder isolates. No resistant breeder isolates were detected in 2017–2019 (Additional file 5). Genotypic resistance to quinolones (*gyrA*) was predicted in 48/188 broiler isolates (25.5%) and resistance to TET (*tet(O)*) in 16/188 broiler isolates (8.5%) (Fig. 1; Table 2, and Additional file 3). The corresponding resistance determinants showed high agreement with the phenotypic resistance data, with sensitivities of 96.0–100.0% and specificities of 99.4–100.0% (Table 2). One ciprofloxacin-resistant isolate and two nalidixic acid-resistant isolates lacked a known resistance-conferring *gyrA* mutation (T86I or T86A), whereas one tetracycline-susceptible (MIC ≤ 0.5 mg/L) isolate carried the *tet(O)* gene.

Notably, in 2014, genotypic resistance to quinolones was more frequent among broiler (16/64, 25.0%) than breeder (4/36, 11.1%) isolates, and a similar pattern was observed for TET resistance: 10/64 (15.6%) versus 3/36 (8.3%) (Additional file 5). However, due to the strategic selection of resistant broiler isolates for WGS, annual genotypic resistance rates in 2015–2019 were partially disparate from the phenotypic resistance rates in the FINRES-Vet data (Additional file 1), and therefore, these results need to be interpreted with caution.

### Distribution of antibiotic resistance determinants among different MLST CCs

Genotypic quinolone resistance was observed altogether in 5/156 ST-45 CC (3.2%), 33/35 ST-206 CC (94.3%), 9/17 ST-21 CC (52.9%), 1/7 ST-952 CC (14.3%), 3/3 ST-658 CC (100%), and 1/1 ST-52 CC (100%) isolates. All breeder isolates with a quinolone-resistant genotype (*n* = 5) harboured the *gyrA* mutation T86I. Of the 48 broiler isolates with a quinolone-resistant genotype, 5 isolates of ST-45 CC had T86A in *gyrA* gene instead of T86I and showed also phenotypic resistance against NAL but not against CIP (Table 2 and Additional file 3). For the isolates with *gyrA* T86A mutation, the MIC [mg/L] against CIP was ≤ 0.5 (S) and against NAL ≥ 32 (mostly 64) (R), in comparison to ≥ 4 (mostly ≥ 16) (R) and > 64 (R) for the broiler isolates (*n* = 43) containing the T86I mutation. One isolate (FIXT-140, ST-45) with phenotypic resistance profile CIP-NAL (MIC > 4 and > 64 mg/L, respectively) and one NAL-resistant isolate (MIC > 64 mg/L) (FIXT-145, ST-11156) lacked a known resistance-conferring mutation in *gyrA*.

Genotypic TET resistance was detected in 14/35 ST-206 CC (40.0%), 2/7 ST-952 CC (28.6%), 2/3 ST-658 CC (66.7%), and 1/1 ST-1034 CC (100%) isolates, as well as in 7 additional isolates representing various STs unassigned to CCs. Among the ten breeder isolates with a TET-resistant genotype, one ST-122 isolate carried the *tet(O/32/O)* variant, whereas the remaining nine isolates carried *tet(O)* and represented various other STs. Phenotypic TET resistance was associated with the *tet(O)* gene in two ST-658 broiler isolates (FIXT-142 and − 143) and with *tet(O/32/O)* in all 13 ST-122 broiler isolates. In contrast, one ST-11,156 broiler isolate (FIXT-145) carried *tet(O)* but was phenotypically susceptible (MIC ≤ 0.5 mg/L) against TET. Further analysis showed that the tetracycline-susceptible isolate (FIXT-145) carried a non-synonymous mutation in *tet(O)* leading to a G → A transition and an associated R623H amino acid change in the encoded tetracycline resistance protein (Additional file 6).

Nearly all breeder (135/145, 93.1%) and broiler (177/188, 94.1%) isolates carried a *bla*_*OXA*_ gene (*-61* [*n* = 172], *-184* [*n* = 16], *-446* [*n* = 31], *-447* [*n* = 65], *-448* [*n* = 12], *-449* [*n* = 11], *-461* [*n* = 1], and *− 465* [*n* = 4]) and were predicted to be resistant against ampicillin (Additional file 3). However, phenotypic ampicillin resistance was not determined. Furthermore, altogether seventeen isolates (5.1%) were predicted to be fully susceptible based on the genes and mutations sought in the ResFinder and PointFinder analyses.

### Core genome MLST (cgMLST) clusters and distribution of antibiotic resistance determinants among clusters

Based on ad *hoc* cgMLST analysis, 54 clusters (2–14 isolates in each cluster) were observed with maximum 11 allelic differences (Additional files 7 and 8). One-third of all clusters (17/54, 31.5%) comprised single-farm replicates sampled within two months, which represented simultaneously raised breeder or broiler flocks, respectively, in different compartments or broiler houses of the same farm. These were mainly susceptible clusters (16/17, 94.1%). All 19 breeder-only clusters showed susceptibility to all the studied antibiotics and were small (harboured 2–4 isolates originating from a maximum of 2 farms). Among 22 clusters comprising only broiler isolates, we observed 17 susceptible clusters, 1 CIP-NAL-TET resistant cluster (representing ST-658 CC), and 4 CIP-NAL resistant clusters (representing ST-21 CC, ST-45 CC, and ST-206 CC).

Thirteen clusters (24.1%) comprised both breeder and broiler isolates, the majority of which were susceptible to antibiotics and represented ST-45 CC or ST-677 CC. Two clusters with both breeder and broiler isolates showed resistance to antibiotics, namely CIP-NAL-TET and CIP-NAL resistance, respectively, representing ST-122 (ST-206 CC) and ST-227 (ST-206 CC). Moreover, almost all of the quinolone-resistant breeder isolates (4/5, 80.0%) were associated with these two resistant clusters, whereas the majority of tetracycline-resistant breeder isolates (9/10, 90.0%) were sporadic findings (i.e. outside clusters). The majority of both quinolone-resistant broiler isolates (40/48, 83.3%) and TET-resistant broiler isolates (15/16, 93.8%) belonged to clusters of either breeder-broiler or broiler-only isolates. In phylogenomic analysis, breeder and broiler isolates were scattered around the tree (Fig. 1). However, one branch abundant with breeder isolates (*n* = 50) was detected, comprising isolates of various STs, mainly unassigned to CCs (Fig. 1).

### Spatiotemporal analysis of cgMLST clusters

In the ad *hoc* cgMLST analysis, seven resistant clusters were identified, each associated with either the CIP-NAL or CIP-NAL-TET resistance profile (Table 3 and Additional file 7). The largest resistant cluster (cluster 1) comprised 14 broiler isolates of ST-2104 (ST-206 CC), which had been sampled mainly in July–August 2018, with one additional isolate in August 2019. These isolates originated from six farms, all located within 38 km in the Ostrobothnia region. The second largest resistant cluster (cluster 2) comprised 14 isolates of ST-122 (ST-206 CC), mainly from broilers, which had been sampled in September–October 2014, June 2015, and February–March 2016. These isolates originated from 13 farms, located within 55 km in Ostrobothnia. The remaining resistant clusters comprised two to five isolates, each sampled within five months from one or two farms.

Interestingly, some of the same farms were involved in resistant clusters 1, 2, 28, and 44 in Ostrobothnia (farms numbered in Additional file 7). The distance between the farms in these resistant clusters belonging to ST-206 CC or ST-21 CC was typically ≤ 55 km. The distance between farms in a resistant cluster belonging to ST-45 CC (cluster 16) was up to 169 km, and one of these farms was located in the same region as clusters 1 and 2. Even longer distances, up to 243 km, were observed between farms in susceptible clusters belonging to ST-45 CC (Additional file 7). Overall, all clustered farms were located within 291 km of each other.

Interestingly, on one occasion the same *C. jejuni* clone was identified in samples collected nearly a year apart from a single breeder farm (cluster 9, farm 26, ST-45), indicating persistence across successive, non-overlapping breeder flock production cycles. More commonly, identical genotypes were detected in slaughter batches separated by only one month (cluster 20, farms 34 and 41, ST-45; and cluster 32, farm 25, ST-230), reflecting concurrent rearing of breeder flocks in different compartments on the same farm rather than sequential production cycles.

In resistant cluster 16, a positive broiler flock on farm 48 was slaughtered one month before the first positive broiler flock on farm 113, located 169 km away. A second positive broiler flock on farm 113 was detected three months later with the same *C. jejuni* clone, consistent with persistence across successive broiler production cycles.

**Table 3:** Antimicrobial-resistant cgMLST clusters identified among 333 *C. jejuni* isolates

Cluster no.	Allele difference	Distance in km between farms, min–max (no. of farms)	ST	CC	Year (no. of isolates)	Source (no. of isolates)	Resistance profile (no. of isolates)	Resistance determinants
1	0 to 2	0–38 (6)	2104	206 CC	2018 (13), 2019 (1)	broiler (14)	CIP-NAL (14)	*gyrA* (T86I), *bla*_*OXA−61*_
2	0 to 8	0–55 (13)	122	206 CC	2014 (10), 2015 (1), 2016 (3)	broiler (13), breeder (1)	CIP-NAL-TET (14)	*gyrA* (T86I), *tet(O/32/O)*, *bla*_*OXA−61*_
5	1 to 5	0–32 (2)	227	206 CC	2014 (5)	broiler (2), breeder (3)	CIP-NAL (5)	*gyrA* (T86I), *bla*_*OXA−61*_
11	6 to 11	0–11 (2)	19	21 CC	2019 (4)	broiler (4)	CIP-NAL (4)	*gyrA* (T86I), *bla*_*OXA−61*_
16	1 to 6	0–169 (2)	230	45 CC	2016 (3)	broiler (3)	CIP-NAL (3)	*gyrA* (T86A), *bla*_*OXA−61*_
28	2	0–0 (1)	19	21 CC	2019 (2)	broiler (2)	CIP-NAL (2)	*gyrA* (T86I), *bla*_*OXA−61*_
44	4	0–0 (1)	658	658 CC	2016 (2)	broiler (2)	CIP-NAL-TET (2)	*gyrA* (T86I), *tet(O)*, *bla*_*OXA−61*_

### Epidemiological links between *C. jejuni* isolates from breeder and broiler flocks

Altogether, 13 breeder isolates shared the same ST with an epidemiologically associated isolate from a descendant broiler flock (Additional file 9). The majority of these breeder isolates (12/13, 92.3%) represented the most predominant ST, ST-45 (ST-45 CC), and were genotypically susceptible to quinolones and TET. One genotypically susceptible breeder isolate (FIXT-1921) represented ST-203 (ST-45 CC). Moreover, the majority of these breeder isolates (8/13, 61.5%) belonged to ad *hoc* cgMLST clusters, namely clusters 3 (FIXT-1841), 9 (FIXT-1911, -1913, and − 1928), 20 (FIXT-1941 and − 1942), 22 (FIXT-1921), and 46 (FIXT-1938) (Additional file 7). However, none of the clusters contained breeder isolates together with epidemiologically associated broiler isolates, contradicting the possibility of vertical transmission.

## Discussion

In the EU, resistance rates of 70.9% against CIP and 50.7% against TET were reported in *C. jejuni* broiler isolates in 2022–2023, whereas markedly lower resistance rates of 1.4% and 0.0%, respectively, were reported in Finland [2]. Nevertheless, during 2014–2019 in Finland, peak annual resistance rates of 8.4–25.5% against CIP and 6.0–17.0% against TET were observed in 2014, 2016, 2018, and 2019, with years of no resistance in between (2015 and 2017) [19]. In this study, higher resistance rates against CIP and TET were observed among *C. jejuni* isolates from broiler flocks than breeder flocks in the peak year 2014. In contrast, in 2015 and 2016, higher genotypic resistance rates (8.7% and 11.9%, respectively) against TET were observed among breeder isolates than reported in the phenotypic FINRES-Vet monitoring (0.0% and 6.0%, respectively) for broiler isolates [19]. Phenotypic and genotypic results were concordant for broiler isolates. Overall, the low and annually fluctuating genotypic resistance observed among breeder isolates and the lack of consistent temporal patterns in resistance between breeder and broiler isolates suggest that breeder flocks have not been a major or consistent source of antimicrobial resistance in Finnish broiler production during the study period. The sporadic detection of resistance among breeder isolates, however, indicates that continued surveillance remains warranted to better understand their potential contribution to resistance dynamics within the production system.

A few dominant genotypic lineages (ST-45 CC, ST-677 CC, and ST-206 CC) were observed among both breeder and broiler isolates, along with diverse sporadic STs. This finding agrees with previous Finnish studies on *C. jejuni* broiler populations, in which ST-45 CC and ST-677 CC were recognized as the most prevalent lineages, along with ST-21 CC [21, 22]. In this study, breeder isolates lacked ST-21 CC and were abundant in one phylogenomic branch, but overall, we observed mainly mixed populations of both breeder and broiler isolates. Previously, Finnish *C. jejuni* broiler isolates from 2014 were shown to differ in whole-genome phylogenies, distribution of STs, and allele frequencies from poultry isolates collected from altogether 12 different European countries [20], indicating that the isolates from Finland were only distantly related to *C. jejuni* populations from other countries. Furthermore, in 2013, the import of grandparent flocks to Finland changed as the import of Hubbard from France replaced Aviagen Ross from Scotland, which had previously been imported to Finland for a long period. In 2014, the import of grandparent flocks ceased and was replaced by the import of parent stock as day-old chicks mainly from Sweden and to a lesser extent from UK (2014) and the Netherlands (2016–2018). In addition, a minority of broiler flocks were imported as hatching eggs from Sweden and Denmark (Hannele Nauholz, personal communication, 3 July 2024). In this study, resistant clusters were associated with broilers from parent stock that originated from various importing countries. A previous comparative genomic study including isolates from France, England, the Netherlands, Sweden, and Denmark demonstrated that Finnish *C. jejuni* populations in 2014 were genetically distinct from those circulating in these countries [20]. Therefore, our results strongly suggest that Finnish breeder isolates represent mainly local populations rather than imported lineages.

In this study, resistance of *C. jejuni* breeder and broiler isolates to quinolones and TET varied markedly between CCs. Among ST-206 CC isolates, 94.3% and 40.0% were resistant to quinolones and TET, respectively, whereas only 3.2% of ST-45 CC isolates were resistant to quinolones and all were susceptible to TET. In addition, 52.9% of ST-21 CC isolates were resistant to quinolones but susceptible to TET. Although formal statistical assessment of the association of resistance with CC was not performed due to biases in the sampling strategy, these patterns were mostly concordant with previous findings. A Belgian study [6] reported that quinolone- and TET-resistant *C. jejuni* isolates from chicken meat were predominantly associated with ST-21 CC, while ST-45 CC isolates were mainly susceptible. Concordantly, an association between ST-21 CC and quinolone resistance has been reported among both clinical and non-human isolates in Central Europe, Egypt, and Chile [5, 8, 9]. Moreover, ST-206 CC, which is genetically closely related to ST-21 CC, has been associated with co-resistance to quinolones and TET in Egypt [9] and Poland [38]. The generally higher antimicrobial susceptibility of ST-45 CC isolates observed in our study was also in line with previous reports, covering data from broiler carcasses and meat in Belgium, as well as humans, dogs, pigs, and cattle in Switzerland [6, 7, 39].

The most commonly reported ciprofloxacin resistance-associated mutation C257T (T86I) in the *Campylobacter gyrA* gene was also detected predominantly in this study. Moreover, the A256G (T86A) mutation was detected but only in five broiler isolates representing ST-45 CC, and it was associated with nalidixic acid resistance but not ciprofloxacin resistance. The common resistance databases, such as ResFinder, predict resistance to fluoroquinolones based on both *gyrA* mutations leading to amino acid changes T86I and T86A, which can be misleading if only genotypic data are used for resistance analyses. Quinolones have neither been used for breeders and broilers in Finland during the collection of data on antibiotic use since 2007 [23], nor before the data collection to our knowledge (Hannele Nauholz, personal communication, 3 July 2024). Therefore, antimicrobial use on the farms cannot explain the spread of resistant clones. Previous research has shown that quinolone resistance induces only minor fitness cost or could even be an advantage for *C. jejuni* in the chicken alimentary canal, which could explain the detection of resistant strains in Finnish poultry farms [40]. Similarly, resistant campylobacter isolates have been detected in poultry in some other countries not using fluoroquinolones for livestock [41].

In this study, over 90% of the isolates carried *bla*_*OXA*_*-*genes. Phenotypic resistance to beta-lactams was, however, not examined, and earlier research has shown that the mere presence of the *bla*_*OXA*_ gene is not directly correlated with beta-lactam resistance [42].

Breeder isolates most commonly harboured the *tet(O)* gene without an association with concurrent fluoroquinolone resistance or certain ST. In contrast, most broiler *C. jejuni* isolates harboured the *tet(O/32/O)* mosaic gene with concurrent C257T (T86I) mutation in *gyrA*. Furthermore, the *tet(O/32/O)* mosaic gene was only detected in isolates representing ST-122 in both breeders (*n* = 1) and broilers (*n* = 13). The *tet(O/32/O)* gene, which encodes a mosaic ribosomal protection protein, has previously been reported in several bacterial species, including *Campylobacter coli* and *C. jejuni*, also in broilers [43–45]. In some years of the study period (2014, 2015, 2016, and 2019), tetracycline was used sporadically to treat breeder flocks during rearing or laying, but not broiler flocks, in Finland (Hannele Nauholz, personal communication, 3 July 2024). This difference in antimicrobial usage may partly explain the observed differences between breeder and broiler *C. jejuni* isolates. In breeders, resistant isolates may have been introduced sporadically from external sources, such as wild birds, and persisted under selection pressure. This is supported by previous studies reporting tetracycline-resistant *C. jejuni* isolates, representing several different STs, from Finnish wild birds [46, 47]. In contrast, the majority of the broiler *C. jejuni* isolates in this study carried the *tet(O/32/O)* variant associated with a single genotype (ST-122, cgMLST cluster 2), further emphasizing the clonality of these ciprofloxacin- and tetracycline-resistant *C. jejuni* isolates from broilers.

One isolate (FIXT-145) in our study carried the *tet(O)* gene yet repeatedly tested phenotypically susceptible to tetracycline. Consequently, resistance would have been overpredicted if inferred solely from genotypic data. Further analysis revealed a non-synonymous point mutation in the *tet(O)* gene that was shown to lead to an amino acid change R623H in the tetracycline resistance protein. We hypothesize that this non-synonymous mutation led to the lack of resistance in this isolate. Future studied are needed to confirm this hypothesis and evaluate the prevalence and persistence of such mutations. To our knowledge, this is the first report of this mutation.

No evidence of vertical transmission was found in this study, although some uncertainty remains. Most broiler flocks consisted of birds originating from several parent flocks, and for many of the broiler flocks, data were missing on epidemiologically linked parent flocks. Also, data were unavailable at the breeder flock level on antibiotic treatments and only partially available on countries of origin. Moreover, both breeder and broiler flocks were sampled only at slaughter, and one isolate per flock was subjected to WGS, thus lacking *C. jejuni* contamination status earlier in the bird’s lifespan and within-flock strain variety. Earlier, a Swedish on-farm study [13] identified different sources and transmission routes of *Campylobacter* colonization of broilers, including nearby cattle, contaminated drinking water, water ponds, transport crates, and parent flocks. They reported similar cgMLST profiles (ST-148) of on-farm broiler isolates and epidemiologically associated parent flock isolates on a single sampling occasion on one farm, suggesting vertical transmission. As discussed earlier [11, 13], little evidence of egg-borne transmission of *Campylobacter* exists, and contamination from a breeder hen could rather be mediated to chicks via faecal contamination of the eggshell. Transmission via the eggshell, however, necessitates the survival of *Campylobacter* in harsh conditions, and especially, combatting dryness [48]. In Finland, eggshells of laid fertile eggs and their transport crates are disinfected mainly using glutaraldehyde fogging at arrival in the hatchery, and no failure of this biosecurity measure could be observed in our results.

Instead of vertical transmission, our results suggest regional spread of resistant *C. jejuni* clones. Clusters of resistant isolates representing ST-206-CC and ST-21 CC were mainly observed in the Ostrobothnia region over one to three consecutive years and involved partly the same or nearby poultry farms. Regional spread further suggests environmental transmission routes between the farms, which remain unidentified from our data collected from slaughterhouse samplings. In the Ostrobothnia region, animal production is concentrated, and further studies should investigate, for instance, the role of nearby cattle and fur animal farms, which have been recognized as potential transmission sources in the spread of resistant *C. jejuni* to broiler farms in previous studies [49, 50]. In contrast to ST-206 CC and ST-21 CC, wider geographical spread was observed within clusters of ST-45 CC, which could imply differences in the environmental fitness and transmission mechanisms of different genotypes. Previous research has suggested that ST-45 CC may survive outside the avian host and contaminate poultry through environmental exposure routes [6].

Although the transmission mechanism between farms warrants further studies, our results highlight the need for enhanced on-farm biosecurity measures since different broiler houses of the same farm were often contaminated by the same *C. jejuni* clone. However, little evidence of transmission between consecutive broiler flocks was found on Finnish farms adhering to an all in – all out principle with cleaning and disinfection between consecutive flocks. In contrast to the all in – all out principle, thinning has been recognized as a major risk factor for *Campylobacter* infections on broiler farms [51]. We therefore recommend sustaining the all in – all out principle to keep the *C. jejuni* prevalence of broiler flocks low in Finland also in future, enhancing the food safety of broiler meat.

## Conclusions

Our findings demonstrate that transient surges in fluoroquinolone-resistant *C. jejuni* in Finnish broiler production were driven by regional clonal spread rather than vertical transmission from breeder flocks. Resistant isolates were strongly associated with specific lineages, particularly ST-206 clonal complex, and showed evidence of localized dissemination among nearby farms. In contrast, genomic analyses provided no evidence for vertical transmission of resistant strains from breeder to broiler flocks. These results highlight the importance of farm-level biosecurity and genomic surveillance for identifying emerging resistant clones and improving control of antimicrobial-resistant *Campylobacter* in poultry production.

## Supplementary Information

Below is the link to the electronic supplementary material.

Supplementary Material 1

Supplementary Material 2

Supplementary Material 3

Supplementary Material 4

Supplementary Material 5

## Data Availability

All data generated or analysed during this study are included in this published article [and its supplementary information files].
