# Supplementary material for: Clonal, regionally clustered spread—rather than vertical transmission—explains surges in fluoroquinolone‑resistant Campylobacter jejuni in Finnish broilers
Source: Acta Vet Scand. 2026 Oct 23;68:46. doi: 10.1186/s13028-026-00887-0 (PMC13695289; doi:10.1186/s13028-026-00887-0)
Supplement: Supplementary file 1 [file 13028_2026_887_MOESM1_ESM.pdf]

Additional file 6. Tet(O) amino acid sequence alignment.

```
CLUSTAL O(1.2.4) multiple sequence alignment


EIA84095.1_Tet[O/32/O]          MKIINLGILAHVDAGKTTLTESLLYTSGAIAELGSVDEGTTRTDTMNLERQRGITIQTAV    60
FIXT-1855_Tet[O/32/O]           MKIINLGILAHVDAGKTTLTESLLYTSGAIAELGSVDEGTTRTDTMNLERQRGITIQTAV    60
HMIK-6032-2014_Tet[O/32/O]      MKIINLGILAHVDAGKTTLTESLLYTSGAIAELGSVDEGTTRTDTMNLERQRGITIQTAV    60
HMIK-6117-2014_Tet[O/32/O]      MKIINLGILAHVDAGKTTLTESLLYTSGAIAELGSVDEGTTRTDTMNLERQRGITIQTAV    60
HMIK-6224-2014_Tet[O/32/O]      MKIINLGILAHVDAGKTTLTESLLYTSGAIAELGSVDEGTTRTDTMNLERQRGITIQTAV    60
HMIK-6249-2014_Tet[O/32/O]      MKIINLGILAHVDAGKTTLTESLLYTSGAIAELGSVDEGTTRTDTMNLERQRGITIQTAV    60
HMIK-6250-2014_Tet[O/32/O]      MKIINLGILAHVDAGKTTLTESLLYTSGAIAELGSVDEGTTRTDTMNLERQRGITIQTAV    60
HMIK-6289-2014_Tet[O/32/O]      MKIINLGILAHVDAGKTTLTESLLYTSGAIAELGSVDEGTTRTDTMNLERQRGITIQTAV    60
HMIK-6512-2014_Tet[O/32/O]      MKIINLGILAHVDAGKTTLTESLLYTSGAIAELGSVDEGTTRTDTMNLERQRGITIQTAV    60
HMIK-6665-2014_Tet[O/32/O]      MKIINLGILAHVDAGKTTLTESLLYTSGAIAELGSVDEGTTRTDTMNLERQRGITIQTAV    60
HMIK-6666-2014_Tet[O/32/O]      MKIINLGILAHVDAGKTTLTESLLYTSGAIAELGSVDEGTTRTDTMNLERQRGITIQTAV    60
FIXT-113_Tet[O/32/O]            MKIINLGILAHVDAGKTTLTESLLYTSGAIAELGSVDEGTTRTDTMNLERQRGITIQTAV    60
FIXT-114_Tet[O/32/O]            MKIINLGILAHVDAGKTTLTESLLYTSGAIAELGSVDEGTTRTDTMNLERQRGITIQTAV    60
FIXT-115_Tet[O/32/O]            MKIINLGILAHVDAGKTTLTESLLYTSGAIAELGSVDEGTTRTDTMNLERQRGITIQTAV    60
HMIK-5967-2014_Tet[O/32/O]      MKIINLGILAHVDAGKTTLTESLLYTSGAIAELGSVDEGTTRTDTMNLERQRGITIQTAV    60
EAQ71799.1_Tet[O]               MKIINLGILAHVDAGKTTLTESLLYTSGAIAELGSVDEGTTRTDTMNLERQRGITIQTAV    60
KRS93435.1_Tet[M]               MKIINLGILAHVDAGKTTLTESLLYTSGAIAELGSVDEGTTRTDTMNLERQRGITIQTAV    60
FIXT-142_Tet[O]                 MKIINLGILAHVDAGKTTLTESLLYTSGAIAELGSVDEGTTRTDTMNLERQRGITIQTAV    60
FIXT-143_Tet[O]                 MKIINLGILAHVDAGKTTLTESLLYTSGAIAELGSVDEGTTRTDTMNLERQRGITIQTAV    60
FIXT-1820_Tet[O]                MKIINLGILAHVDAGKTTLTESLLYTSGAIAELGSVDEGTTRTDTMNLERQRGITIQTAV    60
FIXT-145_Tet[O]                 MKIINLGILAHVDAGKTTLTESLLYTSGAIAELGSVDEGTTRTDTMNLERQRGITIQTAV    60
FIXT-1914_Tet[O]                MKIINLGILAHVDAGKTTLTESLLYTSGAIAELGSVDEGTTRTDTMNLERQRGITIQTAV    60
FIXT-1917_Tet[O]                MKIINLGILAHVDAGKTTLTESLLYTSGAIAELGSVDEGTTRTDTMNLERQRGITIQTAV    60
FIXT-1819_Tet[O]                MKIINLGILAHVDAGKTTLTESLLYTSGAIAELGSVDEGTTRTDTMNLERQRGITIQTAV    60
FIXT-1881_Tet[O]                MKIINLGILAHVDAGKTTLTESLLYTSGAIAELGSVDEGTTRTDTMNLERQRGITIQTAV    60
FIXT-1821_Tet[O]                MKIINLGILAHVDAGKTTLTESLLYTSGAIAELGSVDEGTTRTDTMNLERQRGITIQTAV    60
FIXT-1900_Tet[O]                MKIINLGILAHVDAGKTTLTESLLYTSGAIAELGSVDEGTTRTDTMNLERQRGITIQTAV    60
FIXT-1912_Tet[O]                MKIINLGILAHVDAGKTTLTESLLYTSGAIAELGSVDEGTTRTDTMNLERQRGITIQTAV    60
FIXT-1857_Tet[O]                MKIINLGILAHVDAGKTTLTESLLYTSGAIAELGSVDEGTTRTDTMNLERQRGITIQTAV    60
                                ************************************************************

EIA84095.1_Tet[O/32/O]          TSFQWEDVKVNIIDTPGHMDFLAEVYRSLSVLDGAVLLVSAKDGIQAQTRILFHALQIMK    120
FIXT-1855_Tet[O/32/O]           TSFQWEDVKVNIIDTPGHMDFLAEVYRSLSVLDGAVLLVSAKDGIQAQTRILFHALQIMK    120
HMIK-6032-2014_Tet[O/32/O]      TSFQWEDVKVNIIDTPGHMDFLAEVYRSLSVLDGAVLLVSAKDGIQAQTRILFHALQIMK    120
HMIK-6117-2014_Tet[O/32/O]      TSFQWEDVKVNIIDTPGHMDFLAEVYRSLSVLDGAVLLVSAKDGIQAQTRILFHALQIMK    120
HMIK-6224-2014_Tet[O/32/O]      TSFQWEDVKVNIIDTPGHMDFLAEVYRSLSVLDGAVLLVSAKDGIQAQTRILFHALQIMK    120
HMIK-6249-2014_Tet[O/32/O]      TSFQWEDVKVNIIDTPGHMDFLAEVYRSLSVLDGAVLLVSAKDGIQAQTRILFHALQIMK    120
HMIK-6250-2014_Tet[O/32/O]      TSFQWEDVKVNIIDTPGHMDFLAEVYRSLSVLDGAVLLVSAKDGIQAQTRILFHALQIMK    120
HMIK-6289-2014_Tet[O/32/O]      TSFQWEDVKVNIIDTPGHMDFLAEVYRSLSVLDGAVLLVSAKDGIQAQTRILFHALQIMK    120
HMIK-6512-2014_Tet[O/32/O]      TSFQWEDVKVNIIDTPGHMDFLAEVYRSLSVLDGAVLLVSAKDGIQAQTRILFHALQIMK    120
HMIK-6665-2014_Tet[O/32/O]      TSFQWEDVKVNIIDTPGHMDFLAEVYRSLSVLDGAVLLVSAKDGIQAQTRILFHALQIMK    120
HMIK-6666-2014_Tet[O/32/O]      TSFQWEDVKVNIIDTPGHMDFLAEVYRSLSVLDGAVLLVSAKDGIQAQTRILFHALQIMK    120
FIXT-113_Tet[O/32/O]            TSFQWEDVKVNIIDTPGHMDFLAEVYRSLSVLDGAVLLVSAKDGIQAQTRILFHALQIMK    120
FIXT-114_Tet[O/32/O]            TSFQWEDVKVNIIDTPGHMDFLAEVYRSLSVLDGAVLLVSAKDGIQAQTRILFHALQIMK    120
FIXT-115_Tet[O/32/O]            TSFQWEDVKVNIIDTPGHMDFLAEVYRSLSVLDGAVLLVSAKDGIQAQTRILFHALQIMK    120
HMIK-5967-2014_Tet[O/32/O]      TSFQWEDVKVNIIDTPGHMDFLAEVYRSLSVLDGAVLLVSAKDGIQAQTRILFHALQIMK    120
EAQ71799.1_Tet[O]               TSFQWEDVKVNIIDTPGHMDFLAEVYRSLSVLDGAVLLVSAKDGIQAQTRILFHALQIMK    120
KRS93435.1_Tet[M]               TSFQWEDVKVNIIDTPGHMDFLAEVYRSLSVLDGAVLLVSAKDGIQAQTRILFHALQIMK    120
FIXT-142_Tet[O]                 TSFQWEDVKVNIIDTPGHMDFLAEVYRSLSVLDGAVLLVSAKDGIQAQTRILFHALQTMK    120
FIXT-143_Tet[O]                 TSFQWEDVKVNIIDTPGHMDFLAEVYRSLSVLDGAVLLVSAKDGIQAQTRILFHALQTMK    120
FIXT-1820_Tet[O]                TSFQWEDVKVNIIDTPGHMDFLAEVYRSLSVLDGAVLLVSAKDGIQAQTRILFHALQIMK    120
FIXT-145_Tet[O]                 TSFQWEDVKVNIIDTPGHMDFLAEVYRSLSVLDGAVLLVSAKDGIQAQTRILFHALQIMK    120
FIXT-1914_Tet[O]                TSFQWEDVKVNIIDTPGHMDFLAEVYRSLSVLDGAVLLVSAKDGIQAQTRILFHALQIMK    120
FIXT-1917_Tet[O]                TSFQWEDVKVNIIDTPGHMDFLAEVYRSLSVLDGAVLLVSAKDGIQAQTRILFHALQIMK    120
FIXT-1819_Tet[O]                TSFQWEDVKVNIIDTPGHMDFLAEVYRSLSVLDGAVLLVSAKDGIQAQTRILFHALQIMK    120
FIXT-1881_Tet[O]                TSFQWEDVKVNIIDTPGHMDFLAEVYRSLSVLDGAVLLVSAKDGIQAQTRILFHALQIMK    120
FIXT-1821_Tet[O]                TSFQWEDVKVNIIDTPGHMDFLAEVYRSLSVLDGAVLLVSAKDGIQAQTRILFHALQIMK    120
FIXT-1900_Tet[O]                TSFQWEDVKVNIIDTPGHMDFLAEVYRSLSVLDGAVLLVSAKDGIQAQTRILFHALQIMK    120
FIXT-1912_Tet[O]                TSFQWEDVKVNIIDTPGHMDFLAEVYRSLSVLDGAVLLVSAKDGIQAQTRILFHALQIMK    120
FIXT-1857_Tet[O]                TSFQWEDVKVNIIDTPGHMDFLAEVYRSLSVLDGAVLLVSAKDGIQAQTRILFHALQIMK    120
                                ********************************************************* **

EIA84095.1_Tet[O/32/O]          IPTIFFINKIDQEGIDLPMVYREMKAKLSSEIIVKQKVGQHPHINVTDNDDMEQWDAVIM    180
FIXT-1855_Tet[O/32/O]           IPTIFFINKIDQEGIDLPMVYREMKAKLSSEIIVKQKVGQHPHINVTDNDDMEQWDAVIM    180
HMIK-6032-2014_Tet[O/32/O]      IPTIFFINKIDQEGIDLPMVYREMKAKLSSEIIVKQKVGQHPHINVTDNDDMEQWDAVIM    180
HMIK-6117-2014_Tet[O/32/O]      IPTIFFINKIDQEGIDLPMVYREMKAKLSSEIIVKQKVGQHPHINVTDNDDMEQWDAVIM    180
HMIK-6224-2014_Tet[O/32/O]      IPTIFFINKIDQEGIDLPMVYREMKAKLSSEIIVKQKVGQHPHINVTDNDDMEQWDAVIM    180
HMIK-6249-2014_Tet[O/32/O]      IPTIFFINKIDQEGIDLPMVYREMKAKLSSEIIVKQKVGQHPHINVTDNDDMEQWDAVIM    180
HMIK-6250-2014_Tet[O/32/O]      IPTIFFINKIDQEGIDLPMVYREMKAKLSSEIIVKQKVGQHPHINVTDNDDMEQWDAVIM    180
HMIK-6289-2014_Tet[O/32/O]      IPTIFFINKIDQEGIDLPMVYREMKAKLSSEIIVKQKVGQHPHINVTDNDDMEQWDAVIM    180
HMIK-6512-2014_Tet[O/32/O]      IPTIFFINKIDQEGIDLPMVYREMKAKLSSEIIVKQKVGQHPHINVTDNDDMEQWDAVIM    180
HMIK-6665-2014_Tet[O/32/O]      IPTIFFINKIDQEGIDLPMVYREMKAKLSSEIIVKQKVGQHPHINVTDNDDMEQWDAVIM    180
HMIK-6666-2014_Tet[O/32/O]      IPTIFFINKIDQEGIDLPMVYREMKAKLSSEIIVKQKVGQHPHINVTDNDDMEQWDAVIM    180
```

```
FIXT-113_Tet[0/32/0]         IPTIFFINKIDQEGIDLPMVYREMKAKLSSEIIVKQKVGQHPHINVTDNDDMEQWDAVIM    180
FIXT-114_Tet[0/32/0]         IPTIFFINKIDQEGIDLPMVYREMKAKLSSEIIVKQKVGQHPHINVTDNDDMEQWDAVIM    180
FIXT-115_Tet[0/32/0]         IPTIFFINKIDQEGIDLPMVYREMKAKLSSEIIVKQKVGQHPHINVTDNDDMEQWDAVIM    180
HMIK-5967-2014_Tet[0/32/0]   IPTIFFINKIDQEGIDLPMVYREMKAKLSSEIIVKQKVGQHPHINVTDNDDMEQWDAVIM    180
EAQ71799.1_Tet[0]            IPTIFFINKIDQEGIDLPMVYREMKAKLSSEIIVKQKVGQHPHINVTDNDDMEQWDAVIM    180
KRS93435.1_Tet[M]            IPTIFFINKIDQEGIDLPMVYREMKAKLSSEIIVKQKVGQHPHINVTDNDDMEQWDAVIM    180
FIXT-142_Tet[0]              IPTIFFINKIDQEGIDLPMVYREMKAKLSSEIIVKQKVGQHPHINVTDNDDMEQWEAVIM    180
FIXT-143_Tet[0]              IPTIFFINKIDQEGIDLPMVYREMKAKLSSEIIVKQKVGQHPHINVTDNDDMEQWEAVIM    180
FIXT-1820_Tet[0]             IPTIFFINKIDQEGIDLPMVYREMKAKLSSEIIVKQKVGQHPHINVTDNDDMEQWDAVIM    180
FIXT-145_Tet[0]              IPTIFFINKIDQEGIDLPMVYREMKAKLSSEIIVKQKVGQHPHINVTDNDDMEQWDAVIM    180
FIXT-1914_Tet[0]             IPTIFFINKIDQEGIDLPMVYREMKAKLSSEIIVKQKVGQHPHINVTDNDDMEQWDAVIM    180
FIXT-1917_Tet[0]             IPTIFFINKIDQEGIDLPMVYREMKAKLSSEIIVKQKVGQHPHINVTDNDDMEQWDAVIM    180
FIXT-1819_Tet[0]             IPTIFFINKIDQEGIDLPMVYREMKAKLSSEIIVKQKVGQHPHINVTDNDDMEQWDAVIM    180
FIXT-1881_Tet[0]             IPTIFFINKIDQEGIDLPMVYREMKAKLSSEIIVKQKVGQHPHINVTDNDDMEQWDAVIM    180
FIXT-1821_Tet[0]             IPTIFFINKIDQEGIDLPMVYREMKAKLSSEIIVKQKVGQHPHINVTDNDDMEQWDAVIM    180
FIXT-1900_Tet[0]             IPTIFFINKIDQEGIDLPMVYREMKAKLSSEIIVKQKVGQHPHINVTDNDDMEQWDAVIM    180
FIXT-1912_Tet[0]             IPTIFFINKIDQEGIDLPMVYREMKAKLSSEIIVKQKVGQHPHINVTDNDDMEQWDAVIM    180
FIXT-1857_Tet[0]             IPTIFFINKIDQEGIDLPMVYREMKAKLSSEIIVKQKVGQHPHINVTDNDDMEQWDAVIM    180
                             *******************************************************:****

EIA84095.1_Tet[0/32/0]       GNDELLEKYMSGKPFKMSELEQEENRRFQNGTLFPVYHGSAKNNLGIRQLIEVIASKFYS    240
FIXT-1855_Tet[0/32/0]        GNDELLEKYMSGKPFKMSELEQEENRRFQNGTLFPVYHGSAKNNLGIRQLIEVIASKFYS    240
HMIK-6032-2014_Tet[0/32/0]   GNDELLEKYMSGKPFKMSELEQEENRRFQNGTLFPVYHGSAKNNLGIRQLIEVIASKFYS    240
HMIK-6117-2014_Tet[0/32/0]   GNDELLEKYMSGKPFKMSELEQEENRRFQNGTLFPVYHGSAKNNLGIRQLIEVIASKFYS    240
HMIK-6224-2014_Tet[0/32/0]   GNDELLEKYMSGKPFKMSELEQEENRRFQNGTLFPVYHGSAKNNLGIRQLIEVIASKFYS    240
HMIK-6249-2014_Tet[0/32/0]   GNDELLEKYMSGKPFKMSELEQEENRRFQNGTLFPVYHGSAKNNLGIRQLIEVIASKFYS    240
HMIK-6250-2014_Tet[0/32/0]   GNDELLEKYMSGKPFKMSELEQEENRRFQNGTLFPVYHGSAKNNLGIRQLIEVIASKFYS    240
HMIK-6289-2014_Tet[0/32/0]   GNDELLEKYMSGKPFKMSELEQEENRRFQNGTLFPVYHGSAKNNLGIRQLIEVIASKFYS    240
HMIK-6512-2014_Tet[0/32/0]   GNDELLEKYMSGKPFKMSELEQEENRRFQNGTLFPVYHGSAKNNLGIRQLIEVIASKFYS    240
HMIK-6665-2014_Tet[0/32/0]   GNDELLEKYMSGKPFKMSELEQEENRRFQNGTLFPVYHGSAKNNLGIRQLIEVIASKFYS    240
HMIK-6666-2014_Tet[0/32/0]   GNDELLEKYMSGKPFKMSELEQEENRRFQNGTLFPVYHGSAKNNLGIRQLIEVIASKFYS    240
FIXT-113_Tet[0/32/0]         GNDELLEKYMSGKPFKMSELEQEENRRFQNGTLFPVYHGSAKNNLGIRQLIEVIASKFYS    240
FIXT-114_Tet[0/32/0]         GNDELLEKYMSGKPFKMSELEQEENRRFQNGTLFPVYHGSAKNNLGIRQLIEVIASKFYS    240
FIXT-115_Tet[0/32/0]         GNDELLEKYMSGKPFKMSELEQEENRRFQNGTLFPVYHGSAKNNLGIRQLIEVIASKFYS    240
HMIK-5967-2014_Tet[0/32/0]   GNDELLEKYMSGKPFKMSELEQEENRRFQNGTLFPVYHGSAKNNLGIRQLIEVIASKFYS    240
EAQ71799.1_Tet[0]            GNDELLEKYMSGKPFKMSELEQEENRRFQNGTLFPVYHGSAKNNLGIRQLIEVIASKFYS    240
KRS93435.1_Tet[M]            GNDELLEKYMSGKPFKMSELEQEENRRFQNGTLFPVYHGSAKNNLGTRQLIEVIASKFYS    240
FIXT-142_Tet[0]              GNDELLEKYMSGKPFKMSELEQEENRRFQNGTLFPVYHGSAKNNLGIRQLIEVIASKFYS    240
FIXT-143_Tet[0]              GNDELLEKYMSGKPFKMSELEQEENRRFQNGTLFPVYHGSAKNNLGIRQLIEVIASKFYS    240
FIXT-1820_Tet[0]             GNDELLEKYMSGKPFKMSELEQEENRRFQNGTLFPVYHGSAKNNLGIRQLIEVIASKFYS    240
FIXT-145_Tet[0]              GNDELLEKYMSGKPFKMSELEQEENRRFQNGTLFPVYHGSAKNNLGIRQLIEVIASKFYS    240
FIXT-1914_Tet[0]             GNDELLEKYMSGKPFKMSELEQEENRRFQNGTLFPVYHGSAKNNLGIRQLIEVIASKFYS    240
FIXT-1917_Tet[0]             GNDELLEKYMSGKPFKMSELEQEENRRFQNGTLFPVYHGSAKNNLGIRQLIEVIASKFYS    240
FIXT-1819_Tet[0]             GNDELLEKYMSGKPFKMSELEQEENRRFQNGTLFPVYHGSAKNNLGIRQLIEVIASKFYS    240
FIXT-1881_Tet[0]             GNDELLEKYMSGKPFKMSELEQEENRRFQNGTLFPVYHGSAKNNLGIRQLIEVIASKFYS    240
FIXT-1821_Tet[0]             GNDELLEKYMSGKPFKMSELEQEENRRFQNGTLFPVYHGSAKNNLGIRQLIEVIASKFYS    240
FIXT-1900_Tet[0]             GNDELLEKYMSGKPFKMSELEQEENRRFQNGTLFPVYHGSAKNNLGIRQLIEVIASKFYS    240
FIXT-1912_Tet[0]             GNDELLEKYMSGKPFKMSELEQEENRRFQNGTLFPVYHGSAKNNLGIRQLIEVIASKFYS    240
FIXT-1857_Tet[0]             GNDELLEKYMSGKPFKMSELEQEENRRFQNGTLFPVYHGSAKNNLGIRQLIEVIASKFYS    240
                             ********************************************** *************

EIA84095.1_Tet[0/32/0]       STPEGQSELCGQVFKIEYSEKRRRFVYVRIYSGTLHLRDVIRISEKEKIKITEMCIPSNG    300
FIXT-1855_Tet[0/32/0]        STPEGQSELCGQVFKIEYSEKRRRFVYVRIYSGTLHLRDVIRISEKEKIKITEMCIPSNG    300
HMIK-6032-2014_Tet[0/32/0]   STPEGQSELCGQVFKIEYSEKRRRFVYVRIYSGTLHLRDVIRISEKEKIKITEMCIPSNG    300
HMIK-6117-2014_Tet[0/32/0]   STPEGQSELCGQVFKIEYSEKRRRFVYVRIYSGTLHLRDVIRISEKEKIKITEMCIPSNG    300
HMIK-6224-2014_Tet[0/32/0]   STPEGQSELCGQVFKIEYSEKRRRFVYVRIYSGTLHLRDVIRISEKEKIKITEMCIPSNG    300
HMIK-6249-2014_Tet[0/32/0]   STPEGQSELCGQVFKIEYSEKRRRFVYVRIYSGTLHLRDVIRISEKEKIKITEMCIPSNG    300
HMIK-6250-2014_Tet[0/32/0]   STPEGQSELCGQVFKIEYSEKRRRFVYVRIYSGTLHLRDVIRISEKEKIKITEMCIPSNG    300
HMIK-6289-2014_Tet[0/32/0]   STPEGQSELCGQVFKIEYSEKRRRFVYVRIYSGTLHLRDVIRISEKEKIKITEMCIPSNG    300
HMIK-6512-2014_Tet[0/32/0]   STPEGQSELCGQVFKIEYSEKRRRFVYVRIYSGTLHLRDVIRISEKEKIKITEMCIPSNG    300
HMIK-6665-2014_Tet[0/32/0]   STPEGQSELCGQVFKIEYSEKRRRFVYVRIYSGTLHLRDVIRISEKEKIKITEMCIPSNG    300
HMIK-6666-2014_Tet[0/32/0]   STPEGQSELCGQVFKIEYSEKRRRFVYVRIYSGTLHLRDVIRISEKEKIKITEMCIPSNG    300
FIXT-113_Tet[0/32/0]         STPEGQSELCGQVFKIEYSEKRRRFVYVRIYSGTLHLRDVIRISEKEKIKITEMCIPSNG    300
FIXT-114_Tet[0/32/0]         STPEGQSELCGQVFKIEYSEKRRRFVYVRIYSGTLHLRDVIRISEKEKIKITEMCIPSNG    300
FIXT-115_Tet[0/32/0]         STPEGQSELCGQVFKIEYSEKRRRFVYVRIYSGTLHLRDVIRISEKEKIKITEMCIPSNG    300
HMIK-5967-2014_Tet[0/32/0]   STPEGQSELCGQVFKIEYSEKRRRFVYVRIYSGTLHLRDVIRISEKEKIKITEMCIPSNG    300
EAQ71799.1_Tet[0]            STPEGQSELCGQVFKIEYSEKRRRFVYVRIYSGTLHLRDVIRISEKEKIKITEMYVPTNG    300
KRS93435.1_Tet[M]            STPEGQSELCGQVFKIEYSEKRRRFVYVRIYSGTLHLRDVIRISEKEKIKITEMYVPTNG    300
FIXT-142_Tet[0]              STPEGQSELCGQVFKIEYSEKRRRFVYVRIYSGTLHLRDVIRISEKEKIKITEMCVPTNG    300
FIXT-143_Tet[0]              STPEGQSELCGQVFKIEYSEKRRRFVYVRIYSGTLHLRDVIRISEKEKIKITEMCVPTNG    300
FIXT-1820_Tet[0]             STPEGQSELCGQVFKIEYSEKRRRFVYVRIYSGTLHLRDVIRISEKEKIKITEMCVPTNG    300
FIXT-145_Tet[0]              STPEGQSELCGQVFKIEYSEKRRRFVYVRIYSGTLHLRDVIRISEKEKIKITEMCVPTNG    300
FIXT-1914_Tet[0]             STPEGQSELCGQVFKIEYSEKRRRFVYVRIYSGTLHLRDVIRISEKEKIKITEMCVPTNG    300
FIXT-1917_Tet[0]             STPEGQSELCGQVFKIEYSEKRRRFVYVRIYSGTLHLRDVIRISEKEKIKITEMCVPTNG    300
FIXT-1819_Tet[0]             STPEGQSELCGQVFKIEYSEKRRRFVYVRIYSGTLHLRDVIRISEKEKIKITEMCVPTNG    300
FIXT-1881_Tet[0]             STPEGQSELCGQVFKIEYSEKRRRFVYVRIYSGTLHLRDVIRISEKEKIKITEMCVPTNG    300
```

```
FIXT-1821_Tet[0]                 STPEGQSELCGQVFKIEYSEKRRRFVYVRIYSGTLHLRDVIRISEKEKIKITEMCVPTNG      300
FIXT-1900_Tet[0]                 STPEGQSELCGQVFKIEYSEKRRRFVYVRIYSGTLHLRDVIRISEKEKIKITEMCVPTNG      300
FIXT-1912_Tet[0]                 STPEGQSELCGQVFKIEYSEKRRRFVYVRIYSGTLHLRDVIRISEKEKIKITEMCVPTNG      300
FIXT-1857_Tet[0]                 STPEGQSELCGQVFKIEYSEKRRHFVYVRIYSGTLHLRDVIRISEKEKIKITEMCVPTNG      300
                                 ***********************:****************************** :*:**

EIA84095.1_Tet[0/32/0]           EIVPADHACPGEIVILADDTLKLNDILGNEKLLPHKTRIDNPMPLLRTTVEPQKPEQREA      360
FIXT-1855_Tet[0/32/0]            EIVPADHACPGEIVILADDTLKLNDILGNEKLLPHKTRIDNPMPLLRTTVEPQKPEQREA      360
HMIK-6032-2014_Tet[0/32/0]       EIVPADHACPGEIVILADDTLKLNDILGNEKLLPHKTRIDNPMPLLRTTVEPQKPEQREA      360
HMIK-6117-2014_Tet[0/32/0]       EIVPADHACPGEIVILADDTLKLNDILGNEKLLPHKTRIDNPMPLLRTTVEPQKPEQREA      360
HMIK-6224-2014_Tet[0/32/0]       EIVPADHACPGEIVILADDTLKLNDILGNEKLLPHKTRIDNPMPLLRTTVEPQKPEQREA      360
HMIK-6249-2014_Tet[0/32/0]       EIVPADHACPGEIVILADDTLKLNDILGNEKLLPHKTRIDNPMPLLRTTVEPQKPEQREA      360
HMIK-6250-2014_Tet[0/32/0]       EIVPADHACPGEIVILADDTLKLNDILGNEKLLPHKTRIDNPMPLLRTTVEPQKPEQREA      360
HMIK-6289-2014_Tet[0/32/0]       EIVPADHACPGEIVILADDTLKLNDILGNEKLLPHKTRIDNPMPLLRTTVEPQKPEQREA      360
HMIK-6512-2014_Tet[0/32/0]       EIVPADHACPGEIVILADDTLKLNDILGNEKLLPHKTRIDNPMPLLRTTVEPQKPEQREA      360
HMIK-6665-2014_Tet[0/32/0]       EIVPADHACPGEIVILADDTLKLNDILGNEKLLPHKTRIDNPMPLLRTTVEPQKPEQREA      360
HMIK-6666-2014_Tet[0/32/0]       EIVPADHACPGEIVILADDTLKLNDILGNEKLLPHKTRIDNPMPLLRTTVEPQKPEQREA      360
FIXT-113_Tet[0/32/0]             EIVPADHACPGEIVILADDTLKLNDILGNEKLLPHKTRIDNPMPLLRTTVEPQKPEQREA      360
FIXT-114_Tet[0/32/0]             EIVPADHACPGEIVILADDTLKLNDILGNEKLLPHKTRIDNPMPLLRTTVEPQKPEQREA      360
FIXT-115_Tet[0/32/0]             EIVPADHACPGEIVILADDTLKLNDILGNEKLLPHKTRIDNPMPLLRTTVEPQKPEQREA      360
HMIK-5967-2014_Tet[0/32/0]       EIVPADHACPGEIVILADDTLKLNDILGNEKLLPHKTRIDNPMPLLRTTVEPQKPEQREA      360
EAQ71799.1_Tet[0]                ELYSSDTACSGDIVILPNDVLQLNSILGNEILLPQRKFIENPLPMIQTTIAVKKSEQREI      360
KRS93435.1_Tet[M]                ELYSSDTACSGDIVILPNDVLQLNSILGNEILLPQRKFIENPLPMIQTTIAVKKSEQREI      360
FIXT-142_Tet[0]                  ELYSSDTACSGDIVILPNDVLQLNSILGNEILLPQRKFIENPLPMLQTTIAVKKSEQREI      360
FIXT-143_Tet[0]                  ELYSSDTACSGDIVILPNDVLQLNSILGNEILLPQRKFIENPLPMLQTTIAVKKSEQREI      360
FIXT-1820_Tet[0]                 ELYSSDTACSGDIVILPNDVLQLNSILGNEILLPQRKFIENPLPMLQTTIAVKKSEQREI      360
FIXT-145_Tet[0]                  ELYSSDTACSGDIVILPNDVLQLNSILGNEILLPQRKFIENPLPMLQTTIAVKKSEQREI      360
FIXT-1914_Tet[0]                 ELYSSDTACSGDIVILPNDVLQLNSILGNEILLPQRKFIENPLPMLQTTIAVKKSEQREI      360
FIXT-1917_Tet[0]                 ELYSSDTACSGDIVILPNDVLQLNSILGNEILLPQRKFIENPLPMLQTTIAVKKSEQREI      360
FIXT-1819_Tet[0]                 ELYSSDTACSGDIVILPNDVLQLNSILGNEILLPQRKFIENPLPMLQTTIAVKKSEQREI      360
FIXT-1881_Tet[0]                 ELYSSDTACSGDIVILPNDVLQLNSILGNEILLPQRKFIENPLPMLQTTIAVKKSEQREI      360
FIXT-1821_Tet[0]                 ELYSSDTACSGDIVILPNDVLQLNSILGNEILLPQRKFIENPLPMLQTTIAVKKSEQREI      360
FIXT-1900_Tet[0]                 ELYSSDTACSGDIVILPNDVLQLNSILGNEILLPQRKFIENPLPMLQTTIAVKKSEQREI      360
FIXT-1912_Tet[0]                 ELYSSDTACSGDIVILPNDVLQLNSILGNEILLPQRKFIENPLPMLQTTIAVKKSEQREI      360
FIXT-1857_Tet[0]                 ELYSSDTACSGDIVILPNDVLQLNSILGNEILLPQRKFIENPLPMLQTTIAVKKSEQREI      360
                                 *:  :* ** *:**** :*.*:**.***** ***::. *:**:*:::**:  :* **** 

EIA84095.1_Tet[0/32/0]           LLNALAEIADTDPLLHFDIDTVTHEIMLSFLGNVQMEVICAILEEKYHVEAEIKEPTVIY      420
FIXT-1855_Tet[0/32/0]            LLNALAEIADTDPLLHFDIDTVTHEIMLSFLGNVQMEVICAILEEKYHVEAEIKEPTVIY      420
HMIK-6032-2014_Tet[0/32/0]       LLNALAEIADTDPLLHFDIDTVTHEIMLSFLGNVQMEVICAILEEKYHVEAEIKEPTVIY      420
HMIK-6117-2014_Tet[0/32/0]       LLNALAEIADTDPLLHFDIDTVTHEIMLSFLGNVQMEVICAILEEKYHVEAEIKEPTVIY      420
HMIK-6224-2014_Tet[0/32/0]       LLNALAEIADTDPLLHFDIDTVTHEIMLSFLGNVQMEVICAILEEKYHVEAEIKEPTVIY      420
HMIK-6249-2014_Tet[0/32/0]       LLNALAEIADTDPLLHFDIDTVTHEIMLSFLGNVQMEVICAILEEKYHVEAEIKEPTVIY      420
HMIK-6250-2014_Tet[0/32/0]       LLNALAEIADTDPLLHFDIDTVTHEIMLSFLGNVQMEVICAILEEKYHVEAEIKEPTVIY      420
HMIK-6289-2014_Tet[0/32/0]       LLNALAEIADTDPLLHFDIDTVTHEIMLSFLGNVQMEVICAILEEKYHVEAEIKEPTVIY      420
HMIK-6512-2014_Tet[0/32/0]       LLNALAEIADTDPLLHFDIDTVTHEIMLSFLGNVQMEVICAILEEKYHVEAEIKEPTVIY      420
HMIK-6665-2014_Tet[0/32/0]       LLNALAEIADTDPLLHFDIDTVTHEIMLSFLGNVQMEVICAILEEKYHVEAEIKEPTVIY      420
HMIK-6666-2014_Tet[0/32/0]       LLNALAEIADTDPLLHFDIDTVTHEIMLSFLGNVQMEVICAILEEKYHVEAEIKEPTVIY      420
FIXT-113_Tet[0/32/0]             LLNALAEIADTDPLLHFDIDTVTHEIMLSFLGNVQMEVICAILEEKYHVEAEIKEPTVIY      420
FIXT-114_Tet[0/32/0]             LLNALAEIADTDPLLHFDIDTVTHEIMLSFLGNVQMEVICAILEEKYHVEAEIKEPTVIY      420
FIXT-115_Tet[0/32/0]             LLNALAEIADTDPLLHFDIDTVTHEIMLSFLGNVQMEVICAILEEKYHVEAEIKEPTVIY      420
HMIK-5967-2014_Tet[0/32/0]       LLNALAEIADTDPLLHFDIDTVTHEIMLSFLGNVQMEVICAILEEKYHVEAEIKEPTVIY      420
EAQ71799.1_Tet[0]                LLGALTEISDCDPLLKYYVDTTTHEIILSFLGNVQMEVICAILEEKYHVEAEIKEPTVIY      420
KRS93435.1_Tet[M]                LLGALTEISDCDPLLKYYVDTTTHEIILSFLGNVQMEVICAILEEKYHVEAEIKEPTVIY      420
FIXT-142_Tet[0]                  LLGALTEISDGDPLLKYYVDTTTHEIILSFLGNVQMEVICAILEEKYHVEAEIKEPTVIY      420
FIXT-143_Tet[0]                  LLGALTEISDGDPLLKYYVDTTTHEIILSFLGNVQMEVICAILEEKYHVEAEIKEPTVIY      420
FIXT-1820_Tet[0]                 LLGALKEISDGDPLLKYYVDTTTHEIILSFLGNVQMEVICAILEEKYHVEAEIKEPTVIY      420
FIXT-145_Tet[0]                  LLGALTEISDGDPLLKYYVDTTTHEIILSFLGNVQMEVICAILEEKYHVEAEIKEPTVIY      420
FIXT-1914_Tet[0]                 LLGALTEISDGDPLLKYYVDTTTHEIILSFLGNVQMEVICAILEEKYHVEAEIKEPTVIY      420
FIXT-1917_Tet[0]                 LLGALTEISDGDPLLKYYVDTTTHEIILSFLGNVQMEVICAILEEKYHVEAEIKEPTVIY      420
FIXT-1819_Tet[0]                 LLGALTEISDGDPLLKYYVDTTTHEIILSFLGNVQMEVICAILEEKYHVEAEIKEPTVIY      420
FIXT-1881_Tet[0]                 LLGALTEISDGDPLLKYYVDTTTHEIILSFLGNVQMEVICAILEEKYHVEAEIKEPTVIY      420
FIXT-1821_Tet[0]                 LLGALTEISDGDPLLKYYVDTTTHEIILSFLGNVQMEVICAILEEKYHVEAEIKEPTVIY      420
FIXT-1900_Tet[0]                 LLGALTEISDGDPLLKYYVDTTTHEIILSFLGNVQMEVICAILEEKYHVEAEIKEPTVIY      420
FIXT-1912_Tet[0]                 LLGALTEISDGDPLLKYYVDTTTHEIILSFLGNVQMEVICAILEEKYHVEAEIKEPTVIY      420
FIXT-1857_Tet[0]                 LLGALTEISDGDPLLKYYVDTTTHEIILSFLGNVQMEVICAILEEKYHVEAEIKEPTVIY      420
                                 **.** **:* ****:: :**.****:*********************************

EIA84095.1_Tet[0/32/0]           MERPLRKAEYTIHIEVPPNPFWASVGLSIEPLPIGSGVQYESRVSLGYLNQSFQNAVMEG      480
FIXT-1855_Tet[0/32/0]            MERPLRKAEYTIHIEVPPNPFWASVGLSIEPLPIGSGVQYESRVSLGYLNQSFQNAVMEG      480
HMIK-6032-2014_Tet[0/32/0]       MERPLRKAEYTIHIEVPPNPFWASVGLSIEPLPIGSGVQYESRVSLGYLNQSFQNAVMEG      480
HMIK-6117-2014_Tet[0/32/0]       MERPLRKAEYTIHIEVPPNPFWASVGLSIEPLPIGSGVQYESRVSLGYLNQSFQNAVMEG      480
HMIK-6224-2014_Tet[0/32/0]       MERPLRKAEYTIHIEVPPNPFWASVGLSIEPLPIGSGVQYESRVSLGYLNQSFQNAVMEG      480
HMIK-6249-2014_Tet[0/32/0]       MERPLRKAEYTIHIEVPPNPFWASVGLSIEPLPIGSGVQYESRVSLGYLNQSFQNAVMEG      480
HMIK-6250-2014_Tet[0/32/0]       MERPLRKAEYTIHIEVPPNPFWASVGLSIEPLPIGSGVQYESRVSLGYLNQSFQNAVMEG      480
HMIK-6289-2014_Tet[0/32/0]       MERPLRKAEYTIHIEVPPNPFWASVGLSIEPLPIGSGVQYESRVSLGYLNQSFQNAVMEG      480
```

```
HMIK-6512-2014_Tet[0/32/0]     MERPLRKAEYTIHIEVPPNPFWASVGLSIEPLPIGSGVQYESRVSLGYLNQSFQNAVMEG     480
HMIK-6665-2014_Tet[0/32/0]     MERPLRKAEYTIHIEVPPNPFWASVGLSIEPLPIGSGVQYESRVSLGYLNQSFQNAVMEG     480
HMIK-6666-2014_Tet[0/32/0]     MERPLRKAEYTIHIEVPPNPFWASVGLSIEPLPIGSGVQYESRVSLGYLNQSFQNAVMEG     480
FIXT-113_Tet[0/32/0]           MERPLRKAEYTIHIEVPPNPFWASVGLSIEPLPIGSGVQYESRVSLGYLNQSFQNAVMEG     480
FIXT-114_Tet[0/32/0]           MERPLRKAEYTIHIEVPPNPFWASVGLSIEPLPIGSGVQYESRVSLGYLNQSFQNAVMEG     480
FIXT-115_Tet[0/32/0]           MERPLRKAEYTIHIEVPPNPFWASVGLSIEPLPIGSGVQYESRVSLGYLNQSFQNAVMEG     480
HMIK-5967-2014_Tet[0/32/0]     MERPLRKAEYTIHIEVPPNPFWASVGLSIEPLPIGSGVQYESRVSLGYLNQSFQNAVMEG     480
EAQ71799.1_Tet[0]              MERPLRKAEYTIHIEVPPNPFWASVGLSIEPLPIGSGVQYESRVSLGYLNQSFQNAVMEG     480
KRS93435.1_Tet[M]              MERPLRKAEYTIHIEVPPNPFWASVGLSIEPLPIGSGVQYESRVSLGYLNQSFQNAVMEG     480
FIXT-142_Tet[0]                MERPLRKAEYTIHIEVPPNPFWASVGLSIEPLPIGSGVQYESRVSLGYLNQSFQNAVMEG     480
FIXT-143_Tet[0]                MERPLRKAEYTIHIEVPPNPFWASVGLSIEPLPIGSGVQYESRVSLGYLNQSFQNAVMEG     480
FIXT-1820_Tet[0]               MERPLRKAEYTIHIEVPPNPFWASVGLSIEPLPIGSGVQYESRVSLGYLNQSFQNAVMEG     480
FIXT-145_Tet[0]                MERPLRKAEYTIHIEVPPNPFWASVGLSIEPLPIGSGVQYESRVSLGYLNQSFQNAVMEG     480
FIXT-1914_Tet[0]               MERPLRKAEYTIHIEVPPNPFWASVGLSIEPLPIGSGVQYESRVSLGYLNQSFQNAVMEG     480
FIXT-1917_Tet[0]               MERPLRKAEYTIHIEVPPNPFWASVGLSIEPLPIGSGVQYESRVSLGYLNQSFQNAVMEG     480
FIXT-1819_Tet[0]               MERPLRKAEYTIHIEVPPNPFWASVGLSIEPLPIGSGVQYESRVSLGYLNQSFQNAVMEG     480
FIXT-1881_Tet[0]               MERPLRKAEYTIHIEVPPNPFWASVGLSIEPLPIGSGVQYESRVSLGYLNQSFQNAVMEG     480
FIXT-1821_Tet[0]               MERPLRKAEYTIHIEVPPNPFWASVGLSIEPLPIGSGVQYESRVSLGYLNQSFQNAVMEG     480
FIXT-1900_Tet[0]               MERPLRKAEYTIHIEVPPNPFWASVGLSIEPLPIGSGVQYESRVSLGYLNQSFQNAVMEG     480
FIXT-1912_Tet[0]               MERPLRKAEYTIHIEVPPNPFWASVGLSIEPLPIGSGVQYESRVSLGYLNQSFQNAVMEG     480
FIXT-1857_Tet[0]               MERPLRKAEYTIHIEVPPNPFWASVGLSIEPLPIGSGVQYESRVSLGYLNQSFQNAVMEG     480
                               ************************************************************

EIA84095.1_Tet[0/32/0]         VLYGCEQGLYGWKVTDCKICFEYGLYYSPVSTPADFRLLSPIVLEQALKKAGTELLEPYL     540
FIXT-1855_Tet[0/32/0]          VLYGCEQGLYGWKVTDCKICFEYGLYYSPVSTPADFRLLSPIVLEQALKKAGTELLEPYL     540
HMIK-6032-2014_Tet[0/32/0]     VLYGCEQGLYGWKVTDCKICFEYGLYYSPVSTPADFRLLSPIVLEQALKKAGTELLEPYL     540
HMIK-6117-2014_Tet[0/32/0]     VLYGCEQGLYGWKVTDCKICFEYGLYYSPVSTPADFRLLSPIVLEQALKKAGTELLEPYL     540
HMIK-6224-2014_Tet[0/32/0]     VLYGCEQGLYGWKVTDCKICFEYGLYYSPVSTPADFRLLSPIVLEQALKKAGTELLEPYL     540
HMIK-6249-2014_Tet[0/32/0]     VLYGCEQGLYGWKVTDCKICFEYGLYYSPVSTPADFRLLSPIVLEQALKKAGTELLEPYL     540
HMIK-6250-2014_Tet[0/32/0]     VLYGCEQGLYGWKVTDCKICFEYGLYYSPVSTPADFRLLSPIVLEQALKKAGTELLEPYL     540
HMIK-6289-2014_Tet[0/32/0]     VLYGCEQGLYGWKVTDCKICFEYGLYYSPVSTPADFRLLSPIVLEQALKKAGTELLEPYL     540
HMIK-6512-2014_Tet[0/32/0]     VLYGCEQGLYGWKVTDCKICFEYGLYYSPVSTPADFRLLSPIVLEQALKKAGTELLEPYL     540
HMIK-6665-2014_Tet[0/32/0]     VLYGCEQGLYGWKVTDCKICFEYGLYYSPVSTPADFRLLSPIVLEQALKKAGTELLEPYL     540
HMIK-6666-2014_Tet[0/32/0]     VLYGCEQGLYGWKVTDCKICFEYGLYYSPVSTPADFRLLSPIVLEQALKKAGTELLEPYL     540
FIXT-113_Tet[0/32/0]           VLYGCEQGLYGWKVTDCKICFEYGLYYSPVSTPADFRLLSPIVLEQALKKAGTELLEPYL     540
FIXT-114_Tet[0/32/0]           VLYGCEQGLYGWKVTDCKICFEYGLYYSPVSTPADFRLLSPIVLEQALKKAGTELLEPYL     540
FIXT-115_Tet[0/32/0]           VLYGCEQGLYGWKVTDCKICFEYGLYYSPVSTPADFRLLSPIVLEQALKKAGTELLEPYL     540
HMIK-5967-2014_Tet[0/32/0]     VLYGCEQGLYGWKVTDCKICFEYGLYYSPVSTPADFRLLSPIVLEQALKKAGTELLEPYL     540
EAQ71799.1_Tet[0]              VLYGCEQGLYGWKVTDCKICFEYGLYYSPVSTPADFRLLSPIVLEQALKKAGTELLEPYL     540
KRS93435.1_Tet[M]              VLYGCEQGLYGWKVTDCKICFEYGLYYSPVSTPADFRLLSPIVLEQALKKAGTELLEPYL     540
FIXT-142_Tet[0]                VLYGCEQGLYGWKVTDCKICFEYGLYYSPVSTPADFRLLSPIVLEQALKKAGTELLEPYL     540
FIXT-143_Tet[0]                VLYGCEQGLYGWKVTDCKICFEYGLYYSPVSTPADFRLLSPIVLEQALKKAGTELLEPYL     540
FIXT-1820_Tet[0]               VLYGCEQGLYGWKVTDCKICFEYGLYYSPVSTPADFRLLSPIVLEQALKKAGTELLEPYL     540
FIXT-145_Tet[0]                VLYGCEQGLYGWKVTDCKICFEYGLYYSPVSTPADFRLLSPIVLEQALKKAGTELLEPYL     540
FIXT-1914_Tet[0]               VLYGCEQGLYGWKVTDCKICFEYGLYYSPVSTPADFRLLSPIVLEQALKKAGTELLEPYL     540
FIXT-1917_Tet[0]               VLYGCEQGLYGWKVTDCKICFEYGLYYSPVSTPADFRLLSPIVLEQALKKAGTELLEPYL     540
FIXT-1819_Tet[0]               VLYGCEQGLYGWKVTDCKICFEYGLYYSPVSTPADFRLLSPIVLEQALKKAGTELLEPYL     540
FIXT-1881_Tet[0]               VLYGCEQGLYGWKVTDCKICFEYGLYYSPVSTPADFRLLSPIVLEQALKKAGTELLEPYL     540
FIXT-1821_Tet[0]               VLYGCEQGLYGWKVTDCKICFEYGLYYSPVSTPADFRLLSPIVLEQALKKAGTELLEPYL     540
FIXT-1900_Tet[0]               VLYGCEQGLYGWKVTDCKICFEYGLYYSPVSTPADFRLLSPIVLEQALKKAGTELLEPYL     540
FIXT-1912_Tet[0]               VLYGCEQGLYGWKVTDCKICFEYGLYYSPVSTPADFRLLSPIVLEQALKKAGTELLEPYL     540
FIXT-1857_Tet[0]               VLYGCEQGLYGWKVTDCKICFEYGLYYSPVSTPADFRLLSPIVLEQALKKAGTELLEPYL     540
                               ************************************************************

EIA84095.1_Tet[0/32/0]         HFEIYAPQEYLSRAYHDAPRYCADIVSTQIKNDEVILKGEIPARCIQEYRNDLTCFTNGQ     600
FIXT-1855_Tet[0/32/0]          HFEIYAPQEYLSRAYHDAPRYCADIVSTQIKNDEVILKGEIPARCIQEYRNDLTCFTNGQ     600
HMIK-6032-2014_Tet[0/32/0]     HFEIYAPQEYLSRAYHDAPRYCADIVSTQIKNDEVILKGEIPARCIQEYRNDLTCFTNGQ     600
HMIK-6117-2014_Tet[0/32/0]     HFEIYAPQEYLSRAYHDAPRYCADIVSTQIKNDEVILKGEIPARCIQEYRNDLTCFTNGQ     600
HMIK-6224-2014_Tet[0/32/0]     HFEIYAPQEYLSRAYHDAPRYCADIVSTQIKNDEVILKGEIPARCIQEYRNDLTCFTNGQ     600
HMIK-6249-2014_Tet[0/32/0]     HFEIYAPQEYLSRAYHDAPRYCADIVSTQIKNDEVILKGEIPARCIQEYRNDLTCFTNGQ     600
HMIK-6250-2014_Tet[0/32/0]     HFEIYAPQEYLSRAYHDAPRYCADIVSTQIKNDEVILKGEIPARCIQEYRNDLTCFTNGQ     600
HMIK-6289-2014_Tet[0/32/0]     HFEIYAPQEYLSRAYHDAPRYCADIVSTQIKNDEVILKGEIPARCIQEYRNDLTCFTNGQ     600
HMIK-6512-2014_Tet[0/32/0]     HFEIYAPQEYLSRAYHDAPRYCADIVSTQIKNDEVILKGEIPARCIQEYRNDLTCFTNGQ     600
HMIK-6665-2014_Tet[0/32/0]     HFEIYAPQEYLSRAYHDAPRYCADIVSTQIKNDEVILKGEIPARCIQEYRNDLTCFTNGQ     600
HMIK-6666-2014_Tet[0/32/0]     HFEIYAPQEYLSRAYHDAPRYCADIVSTQIKNDEVILKGEIPARCIQEYRNDLTCFTNGQ     600
FIXT-113_Tet[0/32/0]           HFEIYAPQEYLSRAYHDAPRYCADIVSTQIKNDEVILKGEIPARCIQEYRNDLTCFTNGQ     600
FIXT-114_Tet[0/32/0]           HFEIYAPQEYLSRAYHDAPRYCADIVSTQIKNDEVILKGEIPARCIQEYRNDLTCFTNGQ     600
FIXT-115_Tet[0/32/0]           HFEIYAPQEYLSRAYHDAPRYCADIVSTQIKNDEVILKGEIPARCIQEYRNDLTCFTNGQ     600
HMIK-5967-2014_Tet[0/32/0]     HFEIYAPQEYLSRAYHDAPRYCADIVSTQIKNDEVILKGEIPARCIQEYRNDLTCFTNGQ     600
EAQ71799.1_Tet[0]              HFEIYAPQEYLSRAYHDAPRYCADIVSTQIKNDEVILKGEIPARCIQEYRNDLTYFTNGQ     600
KRS93435.1_Tet[M]              HFEIYAPQEYLSRAYHDAPRYCADIVSTQIKNDEVILKGEIPARCIQEYRNDLTYFTNGQ     600
FIXT-142_Tet[0]                HFEIYAPQEYLSRAYHDAPRYCADIVSTQIKNDEVILKGEIPARCIQEYRNDLTCFTNGQ     600
FIXT-143_Tet[0]                HFEIYAPQEYLSRAYHDAPRYCADIVSTQIKNDEVILKGEIPARCIQEYRNDLTCFTNGQ     600
FIXT-1820_Tet[0]               HFEIYAPQEYLSRAYHDAPRYCADIVSTQIKNDEVILKGEIPARCIQEYRNDLTSFTNGQ     600
FIXT-145_Tet[0]                HFEIYAPQEYLSRAYHDAPRYCADIVSTQIKNDEVILKGEIPARCIQEYRNDLTCFTNGQ     600
FIXT-1914_Tet[0]               HFEIYAPQEYLSRAYHDAPRYCADIVSTQIKNDEVILKGEIPARCIQEYRNDLTCFTNGQ     600
```

```
FIXT-1917_Tet[O]              HFEIYAPQEYLSRAYHDAPRYCADIVSTQIKNDEVILKGEIPARCIQEYRNDLTCFTNGQ      600
FIXT-1819_Tet[O]              HFEIYAPQEYLSRAYHDAPRYCADIVSTQIKNDEVILKGEIPARCIQEYRNDLTCFTNGQ      600
FIXT-1881_Tet[O]              HFEIYAPQEYLSRAYHDAPRYCADIVSTQIKNDEVILKGEIPARCIQEYRNDLTCFTNGQ      600
FIXT-1821_Tet[O]              HFEIYAPQEYLSRAYHDAPRYCADIVSTQIKNDEVILKGEIPARCIQEYRNDLTCFTNGQ      600
FIXT-1900_Tet[O]              HFEIYAPQEYLSRAYHDAPRYCADIVSTQIKNDEVILKGEIPARCIQEYRNDLTCFTNGQ      600
FIXT-1912_Tet[O]              HFEIYAPQEYLSRAYHDAPRYCADIVSTQIKNDEVILKGEIPARCIQEYRNDLTCFTNGQ      600
FIXT-1857_Tet[O]              HFEIYAPQEYLSRAYHDAPRYCADIVSTQIKNDEVILKGEIPARCIQEYRNDLTCFTNGQ      600
                              ****************************************************** *****

EIA84095.1_Tet[O/32/O]        GVCLTELKGYQPAIGKFICQPRRPNSRIDKVRHMFHKLA      639
FIXT-1855_Tet[O/32/O]         GVCLTELKGYQPAIGKFICQPRRPNSRIDKVRHMFHKLA      639
HMIK-6032-2014_Tet[O/32/O]    GVCLTELKGYQPAIGKFICQPRRPNSRIDKVRHMFHKLA      639
HMIK-6117-2014_Tet[O/32/O]    GVCLTELKGYQPAIGKFICQPRRPNSRIDKVRHMFHKLA      639
HMIK-6224-2014_Tet[O/32/O]    GVCLTELKGYQPAIGKFICQPRRPNSRIDKVRHMFHKLA      639
HMIK-6249-2014_Tet[O/32/O]    GVCLTELKGYQPAIGKFICQPRRPNSRIDKVRHMFHKLA      639
HMIK-6250-2014_Tet[O/32/O]    GVCLTELKGYQPAIGKFICQPRRPNSRIDKVRHMFHKLA      639
HMIK-6289-2014_Tet[O/32/O]    GVCLTELKGYQPAIGKFICQPRRPNSRIDKVRHMFHKLA      639
HMIK-6512-2014_Tet[O/32/O]    GVCLTELKGYQPAIGKFICQPRRPNSRIDKVRHMFHKLA      639
HMIK-6665-2014_Tet[O/32/O]    GVCLTELKGYQPAIGKFICQPRRPNSRIDKVRHMFHKLA      639
HMIK-6666-2014_Tet[O/32/O]    GVCLTELKGYQPAIGKFICQPRRPNSRIDKVRHMFHKLA      639
FIXT-113_Tet[O/32/O]          GVCLTELKGYQPAIGKFICQPRRPNSRIDKVRHMFHKLA      639
FIXT-114_Tet[O/32/O]          GVCLTELKGYQPAIGKFICQPRRPNSRIDKVRHMFHKLA      639
FIXT-115_Tet[O/32/O]          GVCLTELKGYQPAIGKFICQPRRPNSRIDKVRHMFHKLA      639
HMIK-5967-2014_Tet[O/32/O]    GVCLTELKGYQPAIGKFICQPRRPNSRIDKVRHMFHKLA      639
EAQ71799.1_Tet[O]             GVCLTELKGYQPAIGKFICQPRRPNSRIDKVRHMFHKLA      639
KRS93435.1_Tet[M]             GVCLTELKGYQPAIGKFICQPRRPNSRIDKVRHMFHKLA      639
FIXT-142_Tet[O]               GVCLTELKGYQPAIGKFICQPRRPNSRIDKVRHMFHKLA      639
FIXT-143_Tet[O]               GVCLTELKGYQPAIGKFICQPRRPNSRIDKVRHMFHKLA      639
FIXT-1820_Tet[O]              GVCLTELKGYQPAIGKFICQPRRPNSRIDKVRHMFHKLA      639
FIXT-145_Tet[O]               GVCLTELKGYQPAIGKFICQPRHPNSRIDKVRHMFHKLA      639
FIXT-1914_Tet[O]              GVCLTELKGYQPAIGKFICQPRRPNSRIDKVRHMFHKLA      639
FIXT-1917_Tet[O]              GVCLTELKGYQPAIGKFICQPRRPNSRIDKVRHMFHKLA      639
FIXT-1819_Tet[O]              GVCLTELKGYQPAIGKFICQPRRPNSRIDKVRHMFHKLA      639
FIXT-1881_Tet[O]              GVCLTELKGYQPAIGKFICQPRRPNSRIDKVRHMFHKLA      639
FIXT-1821_Tet[O]              GVCLTELKGYQPAIGKFICQPRRPNSRIDKVRHMFHKLA      639
FIXT-1900_Tet[O]              GVCLTELKGYQPAIGKFICQPRRPNSRIDKVRHMFHKLA      639
FIXT-1912_Tet[O]              GVCLTELKGYQPAIGKFICQPRRPNSRIDKVRHMFHKLA      639
FIXT-1857_Tet[O]              GVCLTELKGYQPAIGKFICQPRRPNSRIDKVRHMFHKLA      639
                              **********************:****************
```
